# Non-Chromatographic Speciation of As by HG Technique—Analysis of Samples with Different Matrices

**DOI:** 10.3390/molecules25214944

**Published:** 2020-10-26

**Authors:** Maja Welna, Anna Szymczycha-Madeja, Pawel Pohl

**Affiliations:** Department of Analytical Chemistry and Chemical Metallurgy, Faculty of Chemistry, Wroclaw University of Science and Technology, Wybrzeze Wyspianskiego 27, 50-370 Wroclaw, Poland; anna.szymczycha-madeja@pwr.edu.pl (A.S.-M.); pawel.pohl@pwr.edu.pl (P.P.)

**Keywords:** arsenic, arsenic species, hydride generation, atomic spectrometry, non-chromatographic speciation, selective reduction, speciation protocols, environmental, food and biological matrices, sample preparation

## Abstract

The applicability of the hydride generation (HG) sample introduction technique combined with different spectrochemical detection methods for non-chromatographic speciation of toxic As species, i.e., As(III), As(V), dimethylarsinate (DMA) and monomethylarsonate (MMA), in waters and other environmental, food and biological matrices is presented as a promising tool to speciate As by obviating chromatographic separation. Different non-chromatographic procedures along with speciation protocols reported in the literature over the past 20 year are summarized. Basic rules ensuring species selective generation of the corresponding hydrides are presented in detail. Common strategies and alternative approaches are highlighted. Aspects of proper sample preparation before analysis and the selection of adequate strategies for speciation purposes are emphasized.

## 1. Introduction

Because the toxicity and physiological behavior of various As compounds differ greatly, knowledge regarding As species is crucial to understand their potential harmful effects to human beings. Arsenic has a variety of inorganic and organic forms ranging from highly hazardous inorganic arsenicals (i-As), i.e., arsenite (As(III)) and arsenate (As(V)), to relatively less toxic methyl-substituted organic arsenicals (o-As), i.e., monomethylarsonate (MMA) and dimethylarsinate (DMA). Other o-As compounds, e.g., arsenobetaine (AsB), arsenocholine (AsC) and As-sugars, typically present in marine organisms, are generally considered to be non-toxic [1]. Accordingly, today, determination of the total As content is insufficient, and speciation information is essential to reflect the risk associated with exposure to this element. 

As shown in Table 1, contamination with As can be found in various environment compartments including atmosphere (as gaseous compounds and in particulate matter), water, rocks, soil and plants [2,3,4,5,6,7,8,9,10,11,12,13,14,15,16,17,18,19,20,21,22,23,24,25,26,27,28,29,30,31,32,33,34,35,36,37,38,39,40,41,42,43,44,45,46,47,48,49,50,51,52,53,54,55,56,57,58,59]. It can be emitted naturally from volcanic activity or from anthropogenic sources, such as mines, coal-fired plants for energy production, pesticides, phosphate fertilizer factories, irrigation and oxidation of volatile arsines in air, dust from burned fossil fuels as well as the disposal of industrial, municipal and animal waste [32,34]. The widespread use of phosphate rocks in the production of phosphate fertilizers is a significant source of As contamination and cause of exposure to this element [33,36]. The tendency of As to accumulate in plant materials, e.g., cereals, tea plants, vegetables, fruits or herbs, causes it to subsequently appear in food products, beverages or (natural) pharmaceuticals (see Table 1). Among them, rice (together with water) is considered to be the highest contributor to i-As intake among all products of vegetable origin. Although AsB and As-sugars are major components (>90%) in seafood, e.g., fish (AsB) [52,53] and seaweed, e.g., algae (As-sugars) [44,53,54,55], the level of minor i-As, which is responsible for As toxicity in these samples, should also be monitored. Therefore, analyses of the contents of toxic As species in various environmental and food samples would provide information about their quality and safety. On the other hand, analyses of biological/clinical samples like human urine, tissue, cells or blood [4,31,60,61,62,63] would be helpful in recognizing As species in body fluids and tissue to evaluate the pattern of i-As metabolism and assess the risk of adverse health effects associated with i-As exposure. It is worth mentioning that As was also applied historically as a medicinal treatment [56]; nowadays, it is used, e.g., in glass production as a fining agent [35].

Speciation analysis involves two steps, i.e., separation of different forms and their subsequent quantification. The most common and recommended approach providing complete information on the distribution of species and their structures employs chromatographic separation of As species by high performance liquid chromatography (HPLC) interfaced with inductively coupled plasma mass spectrometry (ICP-MS) due to extremely low detection limits (LODs) of As species and their high selectivity [64]. 

Concurrently to HPLC-based As speciation schemes, there is considerable interest in developing simpler, more robust and reliable non-chromatographic methodologies for the determination of toxic As forms in different matrices at trace levels. Among them, hydride generation (HG) is a promising alternative to speciate As by obviating the need for chromatography separation. The generation of volatile hydrides in reactions with sodium/potassium tetrahydroborate (NaBH_4_/KBH_4_) in acidic media (usually HCl) is a well-known derivatization sample introduction technique applied in parallel with atomic and/or mass detection methods for the determination of trace levels of As. It can be easily coupled with various detectors like atomic absorption spectrometry (AAS), atomic fluorescence spectrometry (AFS), inductively coupled plasma optical emission spectrometry (ICP-OES) and ICP-MS, providing selectivity, sensitivity, detectability, separation of analytes from a sample matrix and efficiency that exceeds that offered by conventional pneumatic sample nebulization (PN) [65]. 

The HG technique, originally developed to separate As from the sample matrix, was found to be a viable method by which to discriminate among major toxic As compounds, i.e., i-As, DMA and MMA. Satisfactorily, all these forms are hydride-active and react with NaBH_4_, forming the corresponding hydrides (arsines), i.e., AsH_3_, (CH_3_)_2_AsH and CH_3_AsH_2_ for As(III,V), DMA and MMA [66,67]. Other arseno-organic compounds like AsB and AsC or As-sugars are not reducible and are decomposed to i-As, usually by microwave [51,52] or UV-irradiation, prior to HG [19,68]. However, recent developments have shown that significant HG activity of As-sugars can be achieved under properly chosen conditions in a batch reaction mode [69].

The effectiveness of the HG processes of these individual As forms differs largely and depends strongly on experimental conditions used, i.e., basically, the type and concentration of acid, as well as the pH of the reacting medium. Generally, As(III) can be reduced in a wide HCl concentration range (pH 0–9), while for As(V), strong acidity is required (pH <2) [5,6,70]. In contrast, at low concentrations of HCl, both DMA and MMA hydrides can be effectively generated [71]. Trivalent arsenic can be also converted to arsine in the presence of weak organic acids (citric, acetic, tartaric) [3,40,71,72]. Moreover, this condition could also be applied to HG for DMA and MMA, but not for As(V) [3,71]. Nevertheless, there are considerable differences between the sensitivities obtained for the i-As and o-As forms, and the HG efficiency for V-valent As species is lower than that for As(III) [3,27,38,53,71]. Therefore, finding a compromise reaction medium under which the same response can be obtained for all four As species is problematic. Thus, a pre-reduction step is usually carried out with suitable pre-reductants (mostly KI-ascorbic acid, thiourea and L-cysteine) to ensure that any inorganic and methyl-substituted As(V) forms are present as As(III) before their reaction with NaBH_4_ [3,40,71]. However, similar responses of As(III) and As(V) species under appropriately selected reaction conditions can be achieved, and hence, pre-treatment before HG can be avoided [20,35,72].

On the other hand, the ability of the HG technique to differentiate As forms by their oxidation state (III/V) or nature (inorganic/organic) using simple procedures certainly broadens the application range of HG regarding As speciation, without the use of chromatographic separation (see Table 1). Non-chromatographic approaches to differentiating the four As species are less time-consuming, simpler and more suitable and affordable compared to HPLC [64,73,74]. Generally, selective hydride formation can be achieved by adjusting the reducing conditions in terms of the reaction medium, the NaBH_4_/HCl concentration and the sample pre-treatment with suitable additives. As a result, based on different responses of As species resulting from HG, non-chromatographic protocols for As speciation are proposed with variants for determinations of two- (e.g., [2,12,50,72]), three- (e.g., [7,75,76]) and four- (e.g., [3,28,39,40]) species of As in one sample.

**Figure 1:**
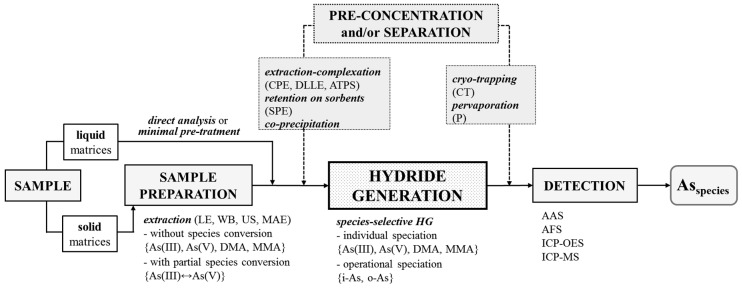
Analytical methodology for non-chromatographic speciation of As by HG technique. AAS: atomic absorption spectrometry. AFS: atomic fluorescence spectrometry. ATPS: liquid-liquid extraction of aqueous two-phase systems. CPE: cloud point extraction. CT: cryogenic cold trapping separation technique. DLLME: dispersive liquid-liquid microextraction. ICP-OES: inductively-coupled plasma optical emission spectrometry. ICP-MS: inductively-coupled plasma mass spectrometry. i-As: the inorganic tri- and penta- valent As species (As(III) and As(V)). LE: solvent extraction. MAE: microwave-assisted extraction. o-As: the organic, i.e., methylated pentavalent As species (DMA and MMA). P: pervaporation-based membrane separation technique. SPE: solid phase extraction. US: ultrasonication in an ultrasound water bath. WB: water bath.

The speciation of As by HG in sample solutions containing various inorganic and organic species is challenging, because several factors affecting the efficiency have to be carefully controlled to improve the accuracy. In reference to this, the present review covers the speciation of As in various matrices including environmental, industrial, food, biological and clinical samples using the non-chromatographic approach, based on the HG technique, and combined with different spectrochemical detection methods. It illustrates the main aspects of proper sample preparation before analysis and the choice of an adequate strategy for speciation (see Figure 1). Analytical methodologies for As speciation by HG are categorized according to their analytical performance, advantages and problems. Different non-chromatographic procedures, along with the speciation protocols reported in the literature over the past 20 year, are summarized. Additionally, the role of specific extraction–complexation, retention and co-precipitation enrichment methods before HG, in addition to the separation of evolved hydrides in cold traps or pervaporation modules to improve the detectability of As species and the selectivity of measurements, are discussed. Finally, alternative methods to conventional wet chemical HG providing selective generation of As hydrides are presented.

## 2. Instrumental Techniques Used for the Determination of As Species and Ways of Verifying the Reliability of the Results

Hydride generation as a sample introduction technique was combined with different sensitive spectrometric detectors to measure the concentration of As species. A great majority of papers cited in the present review are devoted to its application in combination with AAS [3,6,7,12,15,16,18,19,20,24,25,27,33,34,35,36,38,41,46,47,48,50,59,60,61,62,68,70,72,75,76,77,78,79] or AFS [2,5,9,10,13,14,17,21,22,26,28,29,30,32,39,40,42,49,51,52,53,55,56,57,58,63,80,81,82], due to their lower costs in comparison with other techniques, particularly the most expensive ICP-MS, and higher availability in most laboratories. Occasionally, to improve sensitivity and extend the calibration range, ICP-OES is recommended [8,37,45,71,83,84]. Despite the intrinsic advantages of ICP-MS like extremely high sensitivity and a wide dynamic range, i.e., adequate for determination of (ultra)trace quantities of As, this method is rarely applied [11,31,44,53,54].

Generally, the quantification of As was carried out using external calibration with simple standards (e.g., [6,12,15,20,27,28,29,32,33,34,35,36,49,80,83]) or procedural blank-based standard (matrix-matched) solutions (e.g., [2,28,38,44,45,46,47,53,54,59,77]) prepared in the same way as samples (including pre-reduction) to keep the same acidification and the effect of additional reagents used in HG for As species (matrix-matched standards). To account for potential matrix-interfering effects from associated sample constituents, calibration by standard addition was also performed [6,8,17,27,33,34,36,59,72]. The lack of statistically significant differences between the slopes of these calibration curves indicated that there were no interferences, so simple external calibration was acceptable for analysis [6,27,33,34,36,59]. Frequently, to avoid or minimize intensive foam and bubble formation in the gas–liquid separator during HG, usually affecting the As response, silicone-based antifoam emulsions or alcohol-based agents (e.g., n-octanol) were added to the reductant or sample solutions [18,23,27,28,30,40,44,45,46,49,50,53,54,59].

It should be noted that detailed verification of the analytical performance of each method was made by evaluating several quality criteria, including accuracy, (procedural) blanks and analyte-specific figures of merit such as precision (as relative standard deviation, %RSD), LODs and linearity ranges of calibration curves. 

The most reliable approach to demonstrate the accuracy of the whole method of As speciation was based on the analysis of certified reference materials (CRMs). Unfortunately, except for few such CRMs, e.g., NIST 2669 (Human Urine) [4], ERM-BC211 (Rice Flour) [38,46,47,48] and NIST 1568b (Rice Flour) [43], which were developed for As speciation, commercially available CRMs of different matrices with provided certified values of various As species are scare. Nevertheless, other CRMs could be used for As speciation. In this case, the content of As species was analyzed, and then the sum of their concentrations was compared with the certified value of the total As content [10,38,39,44,50,51,52,81]. Similarly, the content of As species in a real sample could also be analyzed, and then the sum of their concentrations could be compared with the total As content obtained after sample decomposition by wet digestion [2,7,40,44,48,49,51]. Due to a lack of proper CRMs, the reliability of the proposed methods was demonstrated by a spike-and-recovery test, based on the addition of known amounts of As species and the application of the whole procedure. Another way to check the accuracy of a newly developed methods is to compare their results with those obtained using well-established methods, e.g., HG-AAS with HPLC-HG-AFS [72], HPLC-ICP-MS [46,79], LC-ICP-MS [7], ETAAS [27] or HG-AFS [59]; HG-AFS with ICP-MS [2,49,50], IC-HG-AFS [28] or HPLC-HG-AFS [5]; HG-ICP-MS with HPLC-(HG)-ICP-MS [44,53,54] or HPLC-HG-AFS [53]. Two different sample preparation procedures, i.e., the reference and the one being examined, followed by measurements using the same detection technique, can also be applied for this purpose, i.e., a slurry sampling versus complete acid digestion in a digest block with a cold finger followed by HG-AAS [33,34], or in a microwave oven (microwave-assisted digestion) followed by ICP-MS [49]. 

## 3. Samples and Their Preparation 

The usefulness of the HG technique for non-chromatographic speciation analysis of As is reflected in a wide spectrum of analyzed samples. This included various environmental, food and biological/clinical materials as follows:

- natural/environmental waters, i.e., mainly drinking [2,3,4,5,14,18,71,83], tap [4,9,15,16,17,18,19,20,21,84,85], ground [4,6,7,8,9,10,11,12,13,14,83], underground [3,15,16], sea [3,10,11,17,24,25,68,80], lake [10,14,20,21,22,23,24,80], river [10,14,20,21,22,75,82,86], waste [4,21] and snow and rain [26]
- sediments [72,75,76,77]
- soil [32,76,77]
- ash [72]
- phosphate rocks [33]
- airborne particular matter [34]
- plants [75]
- agricultural agents, i.e., phosphate fertilizers [33,36], herbicides [37] and pesticides [37]
- glass [35]
- beverages, i.e., alcoholic (wine [27,28]) and alcohol-free (fruit juices [30] and tea [29]),
- cereals, i.e., rice and/or rice products [22,38,41,42,43,44,45,46,47,48] and semolina [39],
- milk [49]
- mushrooms [50]
- vegetables, i.e., eggplant [51], chard [51]
- marine organisms, i.e., seafood [44,52,53,79,81], seaweed/algae [44,53,54,55] and plankton [70]
- pharmaceuticals, i.e., Chinese medicines [56,57,58,78], dietary supplements [59]
- biological fluids/tissue, i.e., human urine [4,62], serum [4], cells [60,61,63] and blood and blood plasma [31]

Unfortunately, to determine the concentrations of particular As species by atomic and/or mass detection methods along with the HG technique, samples need to be in a liquid form. When focusing on speciation analyses, special attention should be paid to the sample preparation step in order to maintain the original characteristics of the species and avoid any changes in a species distribution. 

Sample preparation is unnecessary when analyzing simple liquid samples like water. Usually, before analysis, natural/environmental water samples were only filtered [9,13] through 0.22 [26] or 0.45 μm membrane filters [4,7,10,11,14,16,17,18,19,20,21,23,24,25], or centrifuged [84] to remove suspended solids or insoluble materials. They could be also acidified to 0.01 [CP2] or 0.1 mol L^−1^ HCl [3] or 0.1–1% HNO_3_ [11,16] before [16,23] or after [11] filtering. In some cases, waters were stabilized with concentrated HNO_3_ [6] or HCl [12] immediately after collection. However, the addition of an oxidizing acid would likely lead to erroneous results due to transformations between As(III) and As(V) forms [6], or an interfering effect in the determination of DMA [68]. Occasionally, EDTA [15] or NaF [80] were added to mask interferences in As determination; if precipitation took place afterward, samples were filtered [21]. Samples could also be preserved during storage with a high concentration of an Fe(II) salt, added to slow down the oxidation process of As(III) to As(V) in the presence of microbial activity or oxidizing substances like Fe(III) or Mn(IV) [12,83]. Interestingly, by accomplishing HG with a pervaporation technique used prior to the detection of As species, aqueous “dirty” samples (with a suspended particulate matter) could be analyzed as received, i.e., without any previous filtration [82,86]. Similarly, when detection was performed with the HG technique (not PN), simple cleaning comprising filtration with a 0.45 μm filter was sufficient to yield accurate results for fruit juices [30]. For more complex matrices such as alcoholic beverages, a minimal pre-treatment was required to overcome potential interference from matrix components. However, 20% evaporation of the sample volume [27] or only 5- to 10-fold dilution [28] were enough to completely remove ethanol and get rid of interference during analyses of wines by HG-AAS/AFS. Similarly, liquid rice-based products (wine, beer, vinegar) could simply be diluted (2- to 5-fold) with 0.28 mol L^−1^ HNO_3_ prior to determination of As(III,V) by HG-AAS [47]. A direct dilution pre-treatment with water was found to be adequate to prepare biological/clinical samples such as serum, urine and blood prior to spectrometric measurements combined with HG [4,31,60,61,62,63].

In contrast to liquid samples, As speciation in solid matrices (e.g., food and environmental samples) is a challenge, since all As species must be firstly isolated from the sample matrix before further separation and detection. In contrast, to determine the total As content (usually preceded by wet digestion with aggressive reagents, i.e., commonly concentrated acids), mild but very efficient extraction is required for speciation purposes, ensuring a complete release of As compounds to be determined, albeit with no change in the original identity and concentrations of the individual As species, i.e., oxidation of As(III) to As(V) form and degradation of o-As to i-As forms. Frequently, this was achieved by solvent extraction (LE) with water [70], diluted solutions of HNO_3_ [36,38,40,43,46,52], H_3_PO_4_ [39,50,51] and HCl [33,34,75] or a water:methanol mixture [81]. On the other hand, solvents with partial conversion between As species could also be used. They led to the solubilization of As species, followed by the oxidation of As(III) to As(V), but without decomposition of organic As species to As(V). Typically, extraction and oxidation were carried out in the presence of H_2_O_2_, which was most often used to facilitate the extraction of total i-As species from samples in the form of As(V) [44,53,54]. Agua regia was also found to be effective in this regard [45]. In general, extractions were carried out at elevated temperatures (<100 °C); hence, heating was applied from hot-plates [38,47], shaking water baths (WBs) [41,43] or microwave (MW)-assisted radiation. Nevertheless, MW-assisted extraction (MAE) using conventional MW systems was preferred [38,44,46,53,54,81] to accelerate the release of the analyte into solutions. For As speciation, several procedures were developed based on the application of classical LE. To selectively determine As(III,V) and i-As in rice and rice products by HG-AAS or HG-AFS, extraction with 0.02–0.28 mol L^−1^ HNO_3_ by a WB (90 °C, 60 min) [43], under heating in a WB on a hot plate at 95 °C for 90 min [38,47] or MAE (95 °C for 30 min) [46] was found to be advantageous. Furthermore, in one work [38], the same results as for conventional heating with 0.28 mol L^−1^ HNO_3_ were obtained using MAE (T_max_ 95 °C, 40 min) with 0.14 mol L^−1^ HNO_3_. Otherwise, MAE (50 °C, 5 min) in a water:methanol (1:4, v:v) mixture was the procedure of choice to quantitatively extract all four As species from fish prior to HG-AAS measurements [81]. In cases of selective determination of i-As as As(V) in rice and samples of marine origin (seafood, seaweed), HG-ICP-MS, HG-AAS or HG-AAS, MAE (85–95 °C for 20–40 min) [42,44,53,54,79] or WB extraction (90 °C, 60 min) [41] with diluted HNO_3_-H_2_O_2_ solutions (0.06–0.1 mol L^−1^, 1–3% or 1–2% HNO_3_ combined with 3% H_2_O_2_) were selected. In the case of the extraction procedure with water as the solvent, it was demonstrated that As(III) and As(V) were stable during MAE (800 W, 10 min) of plankton samples, and microwave heating was not able to reduce pentavalent inorganic As [70]. 

More recently, solvent extraction procedures supported by ultrasonic (US) agitation at room temperature (RT), realized using ultrasound WBs [36,39,40,45,50,51,52,75], have been recommended for the speciation of As. However, sample sonication at elevated temperature (80 °C) has also been practiced [40]. Accordingly, regarding individual speciation, a simple procedure for the determination of As(III) and i-As in phosphate fertilizers by HG-AAS was described by Rezende et al. [36], in which samples were sonicated for 35 min with 0.35% Triton X-114 and 6.5 mol L^−1^ HNO_3_. In another work [75], for As speciation in environmental samples (sediment, plant) by HG-AFS, US using 6.0 mol L^−1^ HCl assured the quantitative extraction of As(III), As(V) and DMA; only 10 min was required for sample preparation. Fast US-assisted extraction (10 min) with 1 mol L^−1^ H_3_PO_4_-0.1% Triton XT-100 and of 0.1% EDTA (as a surface cleaning reagent) was also proposed by Gonzalvez et al. [50] to determine As(III) and As(V) by HG-AFS in cultivated and wild mushroom samples of different origins. Similarly, four As species (As(III,V), DMA, MMA) were selectively extracted during 10–20 min sonication of fish [52], cereals [39] and vegetables [51] samples in a mixture of 3 mol L^−1^ HNO_3_ [52] or 1 mol L^−1^ H_3_PO_4_ [39,51] and 0.1% Triton-X114, combined with 0.1% EDTA and analyzed by HG-AFS. Finally, Wang et al. [40] extracted these four target As species from rice by sonication at 80 °C for 10 min with 1% HNO_3_; the whole procedure was repeated three times. US extraction was also advantageous for operational As speciation. Sonication of samples with *aqua regia* for 15 min was used to release four As species from the rice sample matrix, leading to oxidation of As(III) into As(V), but without demethylation of DMA and MMA [45]. i-As species were then determined by HG-ICP-OES. 

A promising excellent alternative to traditional extraction is slurry sampling (SS) based on direct analysis of solid particles of sample dispersed in a liquid phase (mainly diluted acids). Furthermore, it was demonstrated that this sample preparation made it possible to speciate i-As, whereas individual species of As did not undergo any changes in oxidation state. In view of this, recently, some analytical approaches to As speciation in various solid matrices, i.e., airborne particular matter [34], phosphate fertilizers and rocks [33], milk [49] and dietary supplements [59], have been developed, adopting the SS and HG techniques and atomic spectrometry detection. By contrast to traditional LE, Macedo et al. [33,34] employed SS with HG-AAS to determine the total i-As and As(III) in various environmental materials including phosphate fertilizers and rocks [33], and airborne particulate matter samples (filters) [34]. Under optimal conditions, sample portions were mixed with 4.0–6.0 mol L^−1^ HCl and sonicated at RT for 30 min. The resulting sample slurries were diluted with deionized water and further analyzed. Additionally, the bioavailability of As from a breathable particulate matter, which had entered lung fluids, was investigated [34]. In this scenario, deionized water instead of HCl was used as the extracting medium, following the same extraction and quantification methods for the As content. Cava-Montesinos et al. [49] developed a sensitive procedure for the determination of As(III) and As(V) in milk samples by HG-AFS. It was based on the leaching of As species from milk through sonication with *aqua regia* at RT for 10 min, followed by dilution with HCl. Importantly, it was demonstrated that neither As(III) nor As(V) were modified by the proposed sample treatment. This was in contrast to [39,45], where it was reported that the integrity of As(III) and As(V) in *aqua regia* was not preserved, leading to the oxidation of As(III) to As(V) during As species extraction from cereals such as rice [39,45] and wheat semolina [39]. The speciation of inorganic As in various dietary supplements (tablets, capsules) by using the SS and HG-AAS was described by Sun et al. [59], wherein As(III) and As(V) were isolated from the sample matrix during heating with 50% HCl in a WB for boiling for 5–10 min. Since adequate sample slurry preparation is of major importance in this sample technique, critical studies, aiming to achieve stability and uniformity of slurries, were undertaken. The best results were obtained for a sample particle size of 54 μm and a 0.3% agar reagent added to increase the viscosity of the medium. To achieve adequate homogeneity, ultrasonic agitation in an ultrasonic bath at 350 W for 30 min was applied. 

Specific extraction protocols for the determination of inorganic As in ash, sediment and soil were also proposed [32,72,77]. Accordingly, Gonzalez et al. [72] employed a two-step, sequential extraction procedure using water and a 1.0 mmol L^−1^ phosphate buffer to identify As oxidation states in soluble and exchangeable As fractions, respectively, in fly ash and sediment samples. Similarly, Shi et al. [32] introduced a four-step extraction procedure, where deionized water, 0.6 mol L^−1^ KH_2_PO_4_, 1% HCl and 1% NaOH were sequentially used to leach extractable As forms from soils. Solubilized inorganic As compounds were speciated in these extracts by HG-AAS [72] and HG-AFS [32]. Shortened procedures were also proposed to assess the potential bioavailability of toxic As species. Accordingly, Petrov et al. [77] selectively extracted all four As species with EDTA (0.05 mol L^−1^, pH 6–7) from soils and sediments prior to analysis of the EDTA-extractable As fraction by HG-AAS on the content of the sum of As(III,V), DMA and MMA. In another two works [56,57], to understand the solubility, mobility and transport of As in herbaceous plant samples for traditional Chinese medicines (TCMs), as well as commercially available TCMs (herbs), extraction with low concentration HCl [57] and water [56] was recommended. In cases of plants, powdered samples were extracted overnight (RT) with 1% HCl, then filtered and diluted with water. To investigate leachable As species from commercial TCMs, samples were placed in water, heated on a hot-plate to boil for 30 min, and then cooled and filtered. Concentrations of As(III) and total i-As in the obtained sample solutions were measured directly by HG-AFS. Because water is an attractive extracting solution for As, simple brewing, i.e., an everyday culinary process of tea infusion preparation, was also proposed for the speciation of As in tea [29]. For this purpose, a portion of tea was extracted with water for 20 min at 100 °C. Next, a sample suspension was filtered, diluted with water and analyzed for its As(III,V) content by HG-AFS. 

Adequate extraction procedures are crucial for speciation analyses of specific solid samples too. Thus, critical studies were performed by do Nascimento et al. [35] for speciation analysis of As in glass. The authors compared four procedures for glass decomposition in order to determine inorganic As species in various commercial clear, green or amber glass ampoules, bottles and containers. Alkaline fusion with Na_2_CO_3_ (2 h at 900 °C) and glass dissolution in 40% NaOH or diluted HF (24%) for 48 h with or without microwave irradiation (10-50 min at 174 W) were tested. Problems related to either losses of As or stability of As(III) and As(V) forms, as well as incomplete real sample dissolution, were observed using alkaline fusion and the treatment with NaOH/HF supported by heating. Satisfactorily, a cold diluted HF-based strategy was the procedure of choice, and complete recoveries of both As species were obtained.

## 4. Non-Chromatographic As Speciation by Selective HG

The separation of As species in (prepared) sample solutions or sample extracts by selective HG and subsequent spectrometric detection is the most widely applied strategy for non-chromatographic As speciation, combined with the HG technique. Generally, using HG, different reactivities of As(III), As(V), DMA and MMA at various HG reaction conditions are used for the selective generation of individual hydrides. These As hydrides can be generated either selectively at different chemical conditions, or together with other species in various reaction media. Despite these essential advantages, As speciation by HG is focused mainly on i-As species and procedures discriminating between As(III) and As(V) [2,6,12,15,17,20,27,29,32,33,34,35,36,38,47,49,50,56,57,59,72,80,83] or ensuring the selective determination of i-As (as As(III)) [44,45,46,53,54] are those dominating.

### 4.1. Case of Inorganic Arsenic (As(III) and As(V))

#### 4.1.1. Individual Speciation, i.e., Separately Determined As(III) and As(V) 

Several approaches provided by selective HG can be used for the differentiation of As(III) and As(V). Accordingly, species selective HG from As(III,V) is based on:

(a) Different reaction media, i.e., the acidity-dependent reduction reaction between As species and NaBH_4_ to generate hydrides (affected strongly by the type and the concentration of acid or buffer solutions used, as well as the pH of the reacting medium).
(b) The reaction rate with NaBH_4_, i.e., differences in the reduction efficiency between As(III) and As(V) at different NaBH_4_ concentrations (especially low) in the acid medium.
(c) The absence or presence of additives like pre-reduction agents or other specific organic substances including chelating and masking reagents. The addition of various pre-reductants ensures that any As(V) is in the As(III) form before the reaction with NaBH_4_ takes place; this provides the highest sensitivity in the HG technique for As, and hence, makes it possible to quantify the total As content.

Considering the approaches mentioned above, the determination of As(III) and As(V) is quite easy. Generally, a three-step scheme is needed, comprising: (i) the selective determination of As(III) in the presence of As(V); (ii) the determination of total i-As as As(III) after a pre-reduction step for As(V) with a suitable pre-reductant; and finally, (iii) the calculation of the As(V) content as the difference [As(V) = i-As–As(III)]. Accordingly, different reaction media (acids, buffers) [2,6,29,32,33,34,35,36,70,72] and variable concentrations of NaBH_4_ in both soft and highly acidic conditions [12,15,20,27,38,47,72,80,83] were used for the selective determination of As(III) and total i-As. Concurrently to the subtracting method, since responses of As(III) and As(V) in different HG reaction conditions are not the same, linear independent equations relating their analytical signals versus concentrations were also used for quantification [17,29,49,50,56]. The optimum conditions for inorganic As speciation (individual) in various matrices by selective HG and atomic spectrometry detection with LODs of ≤1µg L^−^^1^ are summarized in Table 2. 

It can be concluded that, on the one hand, As(III) reacts selectively with NaBH_4_ in buffered media (with citrates at pH 4.5 [35,36,70,72] and at pH 7.1 [33,34] or Tris-HCl at pH 7.2 [2]) or low concentrated organic acids such as citric acid (0.1 mol L^−1^ [6,32]) or citric acid combined with HCl (0.1 mol L^−1^-5% [29]). On the other hand, selective AsH_3_ formation for As(III) without interference from As(V) can be also achieved via the HG reaction at (very) low NaBH_4_ concentrations (0.035-0.2%) [12,27,38,47,72,80,83], using 0.65–10 mol L^−1^ HCl as the reaction medium. Interestingly, using a batch type HG system, 4% NaBH_4_ combined with 0.1 mol L^−1^ HCl was found to be optimal for direct measurements of As(III) coexisting with As(V) [15]. It should be noted that the As signal increases with the concentration of NaBH_4_; this effect is evident, particularly for As(V). Therefore, the use of low or even very low concentrations of NaBH_4_ decreases the sensitivity of As(III) determination, and hence, the method LOD of As(III) would be affected too [72]. Generally, for this approach, the selected NaBH_4_ concentration is a compromise between the error due to the As(V) interference and the LOD value, making it possible to reliably determine the As(III) content in examined samples [12,83]. Typically, total i-As is determined at higher NaBH_4_ concentrations (0.2–3%) [2,6,12,29,33,34,36,72,83] and 2–10 mol L^−1^ HCl for the HG reaction after the offline pre-reduction of As(V) to As(III) using KI with ascorbic acid [6,12,34,72], thiourea (alone [36] or in combination with ascorbic acid [29]) and L-cysteine [83]. Total i-As can be also determined under the same HG reaction conditions as for As(III) after online [2,32,80] or offline [6,15,27,38,47,70] pre-reduction of As(V) to As(III) with KI alone [6,27], KI-ascorbic acid [15,38,47,80], L-cysteine [32,70] or thioglycolic acid (TGA) [2].

Potassium iodide is one of the most widely used reagents in the pre-reducing step; however, it works effectively only under strong acidic conditions for sample acidification, i.e., 1–11 mol L^−1^ HCl (see e.g., [6,12,13,15,27,34,38,39,45,46,47,49,51,52,71,80]). Sample acidifications of 2–3 mol L^−1^ HCl were the most common [12,13,34,39,45,46,51,52]; nevertheless, lower HCl concentrations in sample solutions (0.1-0.7 mol L^−1^) were also used [3,28]. Typically, ascorbic acid is added along with KI in order to avoid the self-oxidation of I^-^ ions to free I_2_ by the O_2_ that is present in solutions [12,15,46,77,80]. Importantly, the ascorbic acid concentration has no effect on the As signals; thus, its role is primarily to stabilize the pre-reduction medium [80]. Despite this, samples need to be analyzed within 6 h after the addition of reagents to avoid oxidation by atmospheric O_2_, leading to the formation of triiodide and the eventual re-oxidation of As(III) to As(V) [46]. Optionally, to transgress I_2_, sample solutions treated with KI alone can be heated to boil for 5–10 min [59]. Pre-reduction with KI runs commonly for 30–60 min [28,33,34,38,39,44,46,50,51,52,53,59,77], but it can be achieved in a shorter time (i.e., 5 min) by increasing the reagent concentration [80]. By contrast, reagents with thiol (–SH) functional groups such as L-cysteine, thiourea and TGA were found to be more advantageous. Besides its pre-reduction properties, thiourea can also be used as a sensitization reagent, improving the As intensity [56]. L-cysteine pre-reduces all pentavalent As species to their trivalent oxidation state forms with similar responses at a relatively low and narrow optimum HCl concentration range (0.01–0.1 mol L^−1^) [28,71,81]. This reagent was used also to level off the responses of different As species [28,32]. The reaction time for the complete pre-reduction at RT, i.e., 30–60 min [28,52], can be shortened to less than 60 s at 100 °C [52]. Interestingly, pre-reduction with TGA is fast and measurements can be made just after adding this reagents to sample solutions [2,72]. Additionally, it can be used as a pre-reductant for all four As species [2], or can be treated as a reaction medium, making it possible to achieve the same responses with As(III) and As(V) [72].

Gonzalez et al. [72] compared several speciation procedures for the selective determination of As(III) in the presence of As(V) and total i-As by HG-AAS in water-soluble and phosphate-exchangeable extracts of sediments and fly ash CRMs (NIST 1633b, GBW 07311 and GBW 07302). This included (i) AsH_3_ generation under soft HG conditions, i.e., low NaBH_4_ and HCl concentrations (0.05%–2.0%, respectively) and from different reaction media, i.e., citric acid at pH 4.5 and acetic acid for lonely As(III) determination, and (2) total i-As determination in the TGA medium or after pre-reduction with a KI-ascorbic acid mixture. Except for the acetic acid based procedure, all of them could be used to distinguish between these two forms. Acetic acid was not selective enough for the determination of As(III) coexisting with As(V). The best analytical performance was achieved using the procedure with the citric buffer and KI-ascorbic pre-reduction for As(III) and total i-As, respectively. The obtained LODs were two times better than those achieved with the two remaining procedures. On the other hand, in contrast to the pre-reduction of As(V) with KI-ascorbic, the determination of As(III) and As(V) in the presence of TGA medium, with virtually the same responses of both species, could be achieved just after adding this acid to sample solutions. With the KI-ascorbic acid mixture, the conversion of As(V) into As(III) was completed within 1 h. Citric acid was also found to be the most effective reagent in the determination of As(III) alone in soil extracts by HG-AFS [32]. Among the other tested media, including low concentration acetic and tartaric acids, HNO_3_ and HCl, only when citric acid was used, the unwanted presence of As(V) could be virtually eliminated. Similarly, Lehmann et al. [70] succeeded in speciating inorganic As in plankton samples using a metallic furnace atomizer with HG-AAS by controlling the reaction medium and avoiding the reduction of As(V) to As(III). Importantly, measurements of As(III) and total i-As (as As(III) after pre-reduction) were carried out using the same mild conditions for HG, such as a slightly acidic media (a citrate buffer at pH 4.5 was used) and a low NaBH_4_ concentration (0.1%). Among the various pre-reducing agents tested, including KI-ascorbic acid, Na_2_S_2_O_3_, L-cysteine and thiourea, it was found that L-cysteine was able to reduce As(V) to As(III) at pH 4.5. This was probably due to the strong affinity of the As(III) to –SH group present in the L-cysteine structure, which may change the mechanism of reduction and promote the conversion of As(V) to As(III).

Interestingly, in two works [57,59], AsH_3_ was generated selectively for As(III) in the presence of 8-hydroxyquinoline under HCl acidity. Possibly, an ion associate of As(V) with 8-hydroxyquinoline was likely formed, making As(V) unreactive. On the other hand, 8-hydroxyquinoline had an enhancing effect on the responses of both As(III) and As(V) pre-treaded with KI/KI-ascorbic acid before HG. Additionally, the presence of 8-hydroxyquinoline in the reaction medium lessened the interference from transition metals occurring in the generation of arsine. The proposed strategy was found to be attractive in analyses of herbaceous plants by HG-AFS [57] and dietary supplements by HG-AAS [59].

Regarding the different behavior of As(III) and As(V) in the HG process, speciation was also made using proportional equations corresponding to two different measurement conditions for the same sample (acid-NaBH_4_ combination) [17,29,49,50,56]. In three cited works [49,50,56], these two different conditions referred to direct measurements of diluted sample extracts and measurements after the previous pre-reduction step with KI-ascorbic acid-hydroxylamine hydrochloride [49], KI-ascorbic acid [50] or KI-thiourea [56] mixtures. This was successfully applied to determine both i-As species in various materials, including milk [49], mushrooms [50] and TCM herbs [56] by HG-AFS/AAS. To establish these simultaneous equations, two HG reaction conditions combined with L-cysteine for pre-reduction were also used by Wang and Tyson [17] to determine As(III) and As(V) quantities in fresh water samples (sea, tap, pound) by HG-AFS. To overcome interference from Fe(III) in the case of seawater, standard addition calibration curves were employed for As quantification. Finally, Cai et al. [29] obtained a proportional dependence of As species using citric acid for As(III) and thiourea-ascorbic acid for total i-As. The methodology was used to distinguish As(III) and As(V) in tea by HG-AFS.

This subtraction-based speciation scheme could also be simplified and realized without the need of a previous pre-reduction step for total i-As. However, this was possible only for the case when As(III) and As(V) species responded similarly under applied HG conditions. Anthemidis et al. [20] reported that AsH_3_ could be selectively generated either for As(III) or total i-As using different HCl and NaBH_4_ concentrations. As a result, the authors proposed two different pairs of HCl and NaBH_4_ concentrations, i.e., 1.5 mol L^−1^ HCl–0.5% NaBH_4_ and 9.0 mol L^−1^ HCl–3.0% NaBH_4_ for the selective determination of As(III) and total i-As, respectively, in natural water samples (river, lake, tap) by HG-AAS. Similarly, as shown in [35,72], As(III) and As(V) can be determined with the same sensitivity at 0.3% [72] or 1.0% [35] NaBH_4_ in the medium of 0.48% TGA [72] or 6 mol L^−1^ HCl [35] in environmental [72] or glass [35] samples by HG-AAS; no additional pre-reduction step was required.

The main drawback of the proposed speciation schemes could be that the presence of o-As species in the sample matrix, especially MMA and DMA, which generate their respective hydrides under chosen conditions, could selectivity affect the measurements of As(III) and As(V). This is evident when a –SH group containing pre-reductants such as L-cysteine or TGA is used for As(V) pre-reduction. Both result in the same response for all four As species, leading to overestimations of As(V) concentrations [2]. 

In some works, the contribution of methylated As species (o-As) to the quantified i-As concentration was assessed by evaluating the interference of DMA and MMA in the As signal under selected pre-reduction and HG reaction conditions [2,12,17,27,38,47,59,83]. It was found that the increase in HCl concentration (9 mol L^−1^) and the application of KI at the pre-reduction step successfully diminished the negative effects coming from the presence of both DMA and MMA during inorganic As speciation in wine by HG-AAS [27]. In another work [59], the selectivity of As(III) and As(V) measurements in dietary supplements by HG-AAS, i.e., with no interference from either methylated As species, was guaranteed under 1–5% HCl acidity conditions in the presence of 8-hydroxyquinoline and KI, added to pre-reduce As(V) into As(III). In two other works [38,47], using 0.1% NaBH_4_ and 10 mol L^−1^ HCl in combination with KI-ascorbic acid for pre-reduction, it was possible to separately determine As(III) and i-As in rice and rice products by HG-AAS. However, the absence of MMA in the sample was considered; DMA remained undetected, as evidenced by its signal being close to that of the blank. Interference effects coming from DMA and MMA were also noted; therefore, methods developed for the speciation of i-As were proposed and used for samples with negligible or no methylated As compounds [2,12,17,83]. Optionally, to overcome the interference effects from DMA and MMA, solid phase extraction (SPE) with specific sorbents were used to separate i-As from o-As before HG [23,41,42,43,55,79] (see point 5.1). Organic As could also be removed after the HG reaction by freezing out in a liquid nitrogen trap installed between a gas/liquid separator and a detection device [83]. Occasionally, the amount of o-As was defined by subtracting the total i-As content from the total As (As_T_) content determined after complete sample decomposition by wet digestion [27,29,45,50,56,57].

#### 4.1.2. Operational Speciation, i.e., Dealing Only with i-As Determination (total As(III) and As(V)) 

This approach compromises the inorganic As species present in the sample, and is recommended when inter-conversion between As(III) and As(V) is not an issue. The toxicity of As depends on the presence of both inorganic forms; hence, conditions for the selective determination of i-As, i.e., total As(III) and As(V), in the presence of other organoarsenic compounds, including methylated forms and As-sugars, are of special interest.

Depending on the reagents applied at the sample preparation step, e.g., a low concentration of HNO_3_ (alone [46] or combined with H_2_O_2_ [44,53,54]) or *aqua regia* [45]), As can be present in sample extracts in the form of As(III) and As(V) or as i-As, being exclusively As(V) after the conversion of all As(III) to As(V) during extraction. For the selective determination of i-As by HG, i-As species should be present as a single species, i.e., as As(III) or As(V), rather than a mixture of As(III) and As(V). Since As(III) reacts more effectively with NaBH_4_ than As(V), the pre-reduction of As(V) to As(III) prior to HG is preferred. As a result, total i-As is measured in the form of As(III). Selected optimal parameters developed for the selective determination of traces of i-As in rice by HG-ICP-MS [44], HG-ICP-OES [45] or HG-AAS [46], and in more complex matrices, i.e., samples of marine origin such as seaweed, by HG-ICP-MS [44,53,54], are summarized in Table 3.

Generally, the selectivity of i-As determination is based on the application of a high concentration of HCl (5–10 mol L^−1^) for HG and a KI-ascorbic acid mixture for pre-reduction of As(V). By careful selection of flow rates of solutions, a lower optimum HCl concentration (1.2 mol L^−1^) can be achieved [46]. Unfortunately, in a variant of strong sample acidity required for selective HG, L-cysteine could not be used at the pre-reduction step [54]. The concentration of NaBH_4_ used for the determination of i-As varied (0.1–2.0%); nevertheless, its higher concentrations had to be used when HNO_3_ combined with H_2_O_2_ was used to extract both As species. At least 2% NaBH_4_ was needed to overcome the interference effects which occurred in HG for As(V) coming from the presence of H_2_O_2_ in sample extracts left after extraction. Additionally, H_2_O_2_ interfered with pre-reduction of As(V) by KI, which made it impossible to determine the i-As content in real samples in the form of As(III). Accordingly, in these works [44,53,54], total i-As was measured as As(V) after oxidation with H_2_O_2_. In contrast, such an effect was not observed when *aqua regia*- [45] or HNO_3_-based [46] sample extraction procedures were used to release As species from sample matrices.

The main goal of these optimization studies was to provide a contribution of all hydride-active methylated As species to the i-As signal as low as possible. Besides adequate reducing conditions, sample preparation in terms of the reagents used for extraction was helpful in improving the selectivity of i-As determination in the presence of coexisting o-As species. In three works [44,53,54], high concentrations of HCl for HG and H_2_O_2_ in samples of the same concentration, as used for extraction (3%), led to the selective conversion of i-As to volatile arsine, while HG from DMA was substantially inhibited (less than 1-3% of the i-As signal) (see Table 3). Unfortunately, these schemes introduced some errors due to a significant contribution of MMA to the i-As signal (21–43%). However, it is argued that MMA is normally absent or present in trace amounts in most samples of rice and seafood, and hence, would not affect the quantification of i-As in these materials. Interference from a rich As-sugars matrix in the determination of i-As with HG was also negligible; therefore, the described methodologies were suitable as quick reliable screening methods for i-As determination in seaweed [54]. Similar results were achieved for the i-As species measured in sample extracts containing 0.28 mol L^−1^ HNO_3_, used to extract As species from various types of rice (paddy, brown, polished, parboiled) [46]. In one work [45], where *aqua regia* was used for extraction, interferences coming from both DMA and MMA were successfully eliminated, making it possible to reliably differentiate between i-As and o-As, and to selectively determine traces of i-As in brown rice by HG-ICP-OES. The presence of 1.25 mol L^−1^ *aqua regia* in the sample extracts and a high concentration of HCl for the HG reaction were advantageous to limit the activity of o-As during HG, but not i-As.

### 4.2. Case of Inorganic As(III) and As(V) and Organic Arsenic (DMA and MMA)—Speciation and Fractionation Protocols

As presented above, the response of As achievable in the HG reaction strongly depends not only on the oxidation state (III/V) and experimental conditions, but also on the nature of hydride-active As species (inorganic/organic). Accordingly, distinguishing between tri- and penta- valent arsenicals, provided by selective HG, can expand speciation analyses of i-As to its methylated forms (DMA, MMA). Undoubtedly, non-chromatographic approaches to the differentiation of four As species by means of HG are the most desirable, but also the most challenging. Their development has to be proceeded by the careful optimization of experimental parameters, being appropriate for each As species present in the sample solution.

Based on the different reactivities of all four As species under special pre-reducing and HG reaction conditions, procedures for species-selective HG of As can be evaluated using the same rules as those provided for i-As analysis, i.e., pH specific HG reaction or selective conditions (a proper acid-NaBH_4_ combination and a pre-reductant). By combining the responses obtained for these procedures (simple mathematical subtraction or a series of independent proportional equations), protocols for the non-chromatographic speciation of As and determination of its species at a trace level in various matrices, including food [39,40,52,81], beverages [3,7,28] and environmental [3,13,75,76,77] samples, have been proposed. In addition to individual speciation [3,7,28,39,51,52], procedures to fractionate As were also evaluated (operational speciation) by distinguishing between species of the same nature, i.e., i-As versus o-As [71] or As_toxic_ versus As_non-toxic_ [52,71]. The fraction of As_toxic_ indicates hydride-active species (As_h_), i.e., the sum of As(III)+As(V)+DMA+MMA, while the content of As_non-toxic_ refers to unreactive As forms toward HG (As_nh_). Typically, the latter is determined after sample digestion by the difference between the total As content (As_T_) and As_h_. Variants among sensitive determinations of three- and four-species of As are detailed in Table 4.

As shown in Table 4, to selectively generate hydrides for each As species in the reaction with NaBH_4_, the reaction medium—i.e., the type of acid and its acidity, including inorganic (HCl [3,7,13,28,39,51,52,71,75,76,77], HNO_3_ [75]) and carboxylic acids (e.g., tartaric [3,77], acetic [28,71,81], citric [40,71], formic [76], oxalic [77], malic [77]) or buffers (citrate at pH 5 [28,40,71,81]), oxalate at pH 4.5 [7], acetate at pH 4.5 [71]), the concentration of NaBH_4_ [13,28,39,51,52,71], and the kind of the pre-reducing agent employed at the step of the sample pre-treatment (mostly KI/KI-ascorbic acid [3,7,28,39,51,52,71,77,81], thiourea-ascorbic acid [40,71] and L-cysteine [7,28,40,71,81]—was typically controlled. 

Unfortunately, in contrast to the speciation of inorganic As only, one uniform strategy when all four forms are speciated is difficult to establish. However, some selective reaction media for speciation purposes have been recommended. As shown in [28,71,81], which focus on As speciation in wine and fish by HG-AAS [28,81] or drinking water by HG-ICP-OES [71], the determination of only As(III) could be achieved in the presence of a citrate buffer (pH 5), while the presence of a low concentration of acetic acid [28,81] or an acetate buffer (pH 4.5) [71] ensured the generation of arsines for As(III) and DMA. The sum of i-As (As(III) and As(V)) alone [28,81] or together with MMA (i-As+MMA) [71] could be determined after the pre-reduction of V-state As to As(III) with KI/KI-ascorbic and 7–10 mol L^−1^ HCl for the HG reaction. Interestingly, the sum of o-As species, i.e., (DMA+MMA), could be selectively determined after their pre-reduction with L-cysteine, followed by HG in 2 mol L^−1^ HCl [71]. Finally, L-cysteine, used for the pre-reduction of As(V), DMA and MMA to As(III), enabled us to determine total As. Nevertheless, quantitative pre-reduction was reached under completely different HG reaction conditions in terms of the HCl concentration, i.e., 0.01–0.05 mol L^−1^ [28,81] and 10 mol L^−1^ [71]. Otherwise, all four As forms could be speciated by HG-AAS in natural waters (sea, underground, drinking) by conducting the reduction reaction at a fixed NaBH_4_ concentration (0.6%), using different reaction media (HCl, acetic and tartaric acids) and the pre-reduction step with KI [3]. 

In contrast to a popular speciation method by subtraction, it seems that the strategy with HG reaction conditions under which hydrides of As species are generated with different efficiencies may be much easier to establish, as evidenced in several works cited here. In three of them, the determination of all four As forms (As(III,V), DMA and MMA) in fish [52], cereals (rice, wheat semolina) [39] and vegetables (chard, aubergine) [51] by HG-AFS was carried out using a series of independent proportional equations corresponding to four different reduction conditions (I-IV) based on various HCl and NaBH_4_ concentrations, i.e., 2–4 mol L^−1^ HCl and 1.2–1.4% NaBH_4_. An additional pre-treatment with KI-ascorbic acid was applied in one case (condition IV) to reduce As(V) and MMA to As(III). Importantly, non-reducible (non-hydride reactive) As species during HG, such as non-toxic AsB, remained unchanged [51,52]. In the same way, i.e., with the aforementioned linear equation approach, all four As forms were speciated and determined in rice by HG-AFS [40]. It was possible to find four different sample pre-treatment procedures to selectively generate As hydrides in the same HG reaction conditions (1.6 mol L^−1^ HCl-citrate buffer (pH 4.8)-KBH_4_). Several common pre-reducing (KI, L-cysteine, thiourea, ascorbic acid) and preoxidizing (H_2_O_2_, KMnO_4_, K_2_S_2_O_8_) agents that would make it possible to pre-reduce As(V), DMA and MMA to As(III) or oxidize As(III) to As(V) in the sample solution acidified to 0.06 mol L^−1^ citric acid were tested. As a result, in the presence of a low concentration of citric acid alone, a maximum signal for As(III)+DMA was provided (condition I); K_2_S_2_O_8_ completely oxidized As(III), but it did not degrade DMA, and hence, allowed for selective HG for DMA, importantly, without any interference effect from As(V) and MMA (condition II); the use of L-cysteine-ascorbic acid made it possible to determine the sum of As(III), As(V) and DMA, without any contribution of MMA to the overall signal (condition III); finally, the determination of all As species by HG was most effective in the presence of HCl and thiourea-ascorbic acid for pre-reduction (condition IV). It is noteworthy that the results for L-cysteine were similar to those presented in work [70], and proved the pre-reducing potential of this reagent in a citric acid medium (pH 4.5–4.8).

An interesting approach to the determination of the sum of As(III)+DMA+MMA and the sum of all toxicologically relevant hydride-active As species (As(III)+As(V)+DMA+MMA) in EDTA extracts of soil and sediment samples by HG-AAS was evaluated in [77]. An additional pre-treatment with KI-ascorbic acid was applied to reduce As(V) to As(III), and then the As(V) concentration was calculated by the appropriate difference. Moreover, the influence of various types of carboxylic acids, their amino- and hydroxo-derivatives and monosaccharides on the efficiency of the HG process was investigated. Observations showed that EDTA, ascorbic acid, glucose and fructose leveled and equalized the responses of As(III), DMA and MMA at pH 5–7, and furthermore, that ascorbic acid, glucose and fructose maintained their leveling effect at pH 1.3–2 (0.01–0.05 mol L^−1^ HCl).

In two other works [13,76], sample pre-treatment with UV irradiation resulted in photo-oxidation or photo-reduction processes that promoted the inter-conversion of As species prior to HG. Chaparro et al. [13] proposed an approach for the selective determination of total i-As and DMA in ground water samples using an automated HG-AFS system. In the first procedure, total As (the sum of As(III), As(V) and DMA) was determined after UV irradiation of the sample in a K_2_S_2_O_8_ medium and after photo-oxidation of all As species to inorganic As(V). In the second one, total i-As was measured directly (i.e., the photo-oxidation step was omitted) after the previous pre-reduction of As(V) to As(III) with KI-ascorbic acid. The DMA concentration was calculated by the difference. Pinheiro et al. [76] reported that all three As forms were speciated and determined by HG-AAS by coupling a photo-reduction system with a HG manifold. The effective photo-reduction of DMA and As(V) to inorganic As(III) was achieved by using UV treatment (catalyzed by ZnO nanoparticles) in a formic acid medium. Various reaction conditions, in terms of the HCl concentration, were used for the selective generation of individual hydrides. With 2% HCl for the HG reaction, As(III) was directly determined, while As(III)+DMA could be determined after the photo-reduction step. By applying 10% HCl and the same photo-reduction process, As(III)+As(V)+DMA were quantified. The proposed strategies were suitable for As speciation in environmental samples such as water and soil or sediments (after US-assisted extraction). 

However, Pinheiro et al. [75] and Akter et al. [7] demonstrated that As(III), As(V) and DMA could be also selectively determined without UV irradiation of samples. Accordingly, the selective generation of As(III), As(V) and DMA hydrides was possible using 1.2% NaBH_4_ and changing the acid type and its concentration [75]. It is noteworthy that no additional pre-reducing step was required. For each species, different experimental reduction conditions, based on different HCl and HNO_3_ concentrations, were used. Four speciation procedures were developed and their reliability was verified. Employing various HCl concentrations, i.e., 2% and 10%, As(III) and i-As (As(III)+As(V)) were determined, respectively. The content of As(V) was obtained by calculating the difference. Employing various HNO_3_ concentrations, i.e., 15% and 2%, As(III) and As(III)+DMA, were determined, respectively. Similarly to As(V), by the difference in responses found for 2 and 15% HNO_3_, the exact content of DMA was calculated. The proposed speciation approach was applicable for As speciation in sediment and plant samples by HG-AFS. Akter et al. [7] used a well-known procedure for i-As speciation based on selective As(III) determination at pH 4.5 (oxalate buffer) and total i-As in the HCl medium (6 mol L^−1^) after pre-reduction of As(V) to As(III) with KI. Otherwise, the reaction medium of 1.5 mol L^−1^ HCl and the sample pre-treatment with L-cysteine allowed made it possible to determine the DMA alone. The same NaBH_4_ concentration (0.6%) was used in all three procedures. The developed methodology was applied for As speciation in ground water samples by HG-AAS. 

## 5. Pre-Concentration and/or Separation 

The concentrations of As in environmental, food and biological samples are usually very low (see Table 1). The HG technique, coupled with atomic spectrometric detectors, is a sensitive analytical tool for the determination of traces of As. However, when handling ultratrace amounts of As, additionally in complex sample matrices, the direct determination of As species is difficult, and therefore, preliminary pre-treatment comprising the separation and/or pre-concentration of these species is highly desirable. Accordingly, in the sample preparation step, one or more As species (typically inorganic) can be separated through different extraction techniques. Early evolved arsines, both inorganic and methylated, can also be isolated according to their boiling points with the cryo-trapping (CT) technique or by the pervaporation-based membrane separation technique. Both approaches provide an excellent improvement in LODs of As species to ≤ ng L^−^^1^ levels for the same detector and improve the selectivity of measurements due to the alleviation of interference from sample matrix components. 

### 5.1. Pre-Concentration and Separation Methods before HG Process 

Selective complexation–extraction techniques, selective retention on solid adsorbents, i.e., solid phase extraction (SPE), and selective co-precipitations are the most common methods to separate/pre-concentrate As species, mainly inorganic forms, used in the sample preparation stage. Accordingly, As(III) or As(V) can be selectively extracted or co-precipitated with subsequent HG and detection. Total i-As is obtained after the initial conversion of As(III)↔As(V), while the content of As(III) or As(V) can then be calculated by the difference. Non-chromatographic schemes for As speciation with the most popular pre-concentration/separation methods before HG, along with the achieved analytical performance, are summarized in Table S1.

#### 5.1.1. Selective Complexation–Extraction

Among the various extraction techniques that could easily be adapted as the initial step in species-selective and -sensitive As determination combined with HG and spectrometric detection, cloud point extraction (CE), dispersive liquid-liquid microextraction (DLLME) and liquid–liquid extraction of aqueous two-phase systems (ATPS) are very popular.

Cloud point extraction, based on nonionic surfactants used as extracting solvents, offers many advantages like simplicity, safety, low cost and high pre-concentration factors. Different extraction procedures, making it possible to distinguish between i-As species, were proposed in methods developed to monitor As(III) and As(V) in natural water samples (drinking, tap, lake). For example, in one work [18], a selective complex of As(III) with Pyronine B in the presence of sodium dodecyl sulfate (SDS) at pH 10 was extracted using a nonionic surfactant, Triton X-114. The surfactant-rich phase with As(III) was then separated and diluted with 1 mol L^−1^ HCl prior to its determination by HG-AAS. Total i-As (As(III,V)) was extracted similarly after the pre-reduction of As(V) to As(III) with Na_2_S_2_O_3_, and the As(V) content was calculated by the difference. In another work [23], As(III) and As(V) were separated by complexing with ammonium pyrrolidinedithiocarbamate (APDC) (at pH 4.6) and molybdate (at pH 2.4), respectively, followed by quantitative extraction with Triton X-114. Afterwards, the As(III) content was determined by HG-AAS after diluting the surfactant-rich phase with 5% HCl. In the case of As(V), the resulting As(V) complex was first converted to free As(V) by ultrasonication, pre-reduced to As(III) with a thiourea-ascorbic acid mixture, and finally, determined by HG-AAS. 

Dispersive liquid–liquid microextraction aims to extract the analytes from an aqueous phase into an organic phase, in which a third solvent is rapidly injected to accelerate efficient dispersion. Compared to traditional LE, it presents advantages in costs, labor, solvent consumption and enrichment factors. Chen et al. [30] applied the DLLME approach to speciate and quantify As(III) and As(V) in fruit juices in the presence of o-As (DMA and MMA) by HG-AFS using APDC (complexing agent), methanol (dispersant) and CCl_4_ (extractant). Samples were adjusted to pH 3 and then mixed with APDC to form the As(III)-APDC complex, followed by injection of methanol and CCl_4_ to form a dispersion. After centrifugation, the organic phase with the As(III)-APDC complex was evaporated to dryness, and then the residue was dissolved in 1 mol L^−1^ HCl and subjected to analysis by HG-AFS. Total i-As was determined after the pre-reduction of As(V) to As(III) using Na_2_S_2_O_3_; next, the same protocol as for As(III) was used. Finally, the As(V) content was calculated from the difference. Under the selected pre-reduction and HG reaction conditions, limitations of the method for DMA were found to be advantageous to selectively measure i-As. In contrast, MMA contributed to the As response for i-As. However, the degree of this interference was pH-dependent; at pH 1.7–1.8, the error was <10%.

A recent alternative to LE extraction is extraction by the aqueous two-phase system (ATPS). Its advantages include simplicity, low cost and high enrichment factors. These aqueous two-phase systems are primarily composed of water and other compounds of low toxicity. They can be formed by two heterogeneous phases composed of aqueous solutions of two incompatible polymers, i.e., a polymer and an electrolyte, or two types of physically incompatible electrolytes. Assis et al. [84] studied ATPS extraction comprising a polymer and an electrolyte prior to speciation of i-As forms in tap waters by HG-ICP-OES. The authors developed a procedure for the selective extraction of As(III) coexisting with As(V) using the ATPS composed of L64 (copolymers), water and Na_2_SO_4_ at pH 6.0, while APDC was used to extract As(III); As(V) was poorly extracted in these conditions (<18%).

#### 5.1.2. Selective Retention—Solid Phase Extraction

Solid phase extraction is the most popular and commonly applied separation/pre-concentration technique. In SPE, analytes are extracted by sorption, eluted with a small amount of a solvent and then directly detected. Speciation analysis is realized by selective sorption or selective elution. SPE can fulfill the separation/pre-concentration requirements of As species before HG and spectrometric detection using a variety of sorbents. These could be conventional substances such as resins or gels (whose analytical capabilities could be further modified by surface modifications (functionalization) to improve the absorption performance), as well as alternative and novel ones like nanometer-sized materials or biosorbents with exceptional properties to effectively separate specific As species. Moreover, the method can easily be combined with different detection techniques in online [9,10,11,25,26,37,55] or offline modes [4,8,14,16,21,41,43,79].

The selection of appropriate sorbents is a key parameter in SPE. The development of alternative/novel sorbents is a general trend in pre-concentration and separation procedures for As speciation, whereas methodologies aimed at separating arsenate from arsenite are more widespread. Deng et al. [14] developed a simple and rapid method for the determination of trace amounts of total i-As in environmental water samples (ground, river, lake) by SPE on an aluminum hydroxide gel and HG-AFS detection. Trivalent arsenic was first oxidized to As(V) by KMnO_4_; then, the sample solution was adjusted to pH 6, followed by the addition of a freshly prepared gel for extraction of As(V). After centrifugation, the resultant precipitate with adsorbed i-As was dissolved in concentrated HCl; then, As(V) was pre-reduced with a thiourea-ascorbic acid mixture, and finally, total i-As was determined as As(III) by HG-AFS. In two other works, SPE columns packed with cigarette filters [9] or PTFE particles [25] were used prior to HG and determination of the i-As content in various water samples (including tap, ground and seawater). The developed methods were based on selective online formation of the As(III)-APDC complex and its retention on SPE columns. As(V) did not form any complexes with APDC, and could not be retained in these conditions; hence, it passed through the columns. After reducing As(V) to As(III) with L-cysteine [9] or thiourea [25], the same system was applied to determine the total i-As, and As(V) was calculated by the difference. The adsorbed As(III)-APDC complex was online removed from SPE columns using HCl (1.7-2.0 mol L^−1^) and merged with KBH_4_ (2.1%) [9] or NaBH_4_ (4.1%) [25] solutions to generate AsH_3_ before entering AFS [9] or AAS [25] spectrometers for As(III) detection. Importantly, diluted HCl was used as the eluent because it also provided a favorable medium for the HG reaction.

In a few recent studies, graphene or carbon nanotubes have been used as sorbents for SPE. Considering As speciation, Khaligh et al. [4] presented an interesting approach to the speciation of i-As using nonporous graphene functionalized with carboxyl groups (G-COOH) for the ultrasound assisted, dispersive, micro-solid phase extraction (US-D-μ-SPE) of As(V) from several natural water (tap, drinking, river, waste) and biological (human serum/urine) samples prior to its determination by HG-AAS. Briefly, As(V) was selectively retained on the G-COOH sorbent at pH 3.5 by US-D-μ-SPE with the next separation of the solid phase being achieved through centrifugation. Then, the As(V) retained on the sorbent was eluted with NaOH (0.3–0.5 mol L^−1^), pre-reduced to As(III) with a KI-ascorbic acid mixture, and determined by HG-AAS. The previous oxidation of As(III) using KMnO_4_ made it possible to determine the total i-As. The difference between total i-As and As(V) yielded the As(III) content in the analyzed samples. The application of the carbon nanotube (CNT) sorbents for the determination of As(III) and As(V) at (ultra)trace levels in various environmental water samples (rain, snow, sea, river) is demonstrated in [10,26]. Wu et al. [10] packed a micropipette with single-walled (SW) CNTs to achieve the selective adsorption of the As(III)-APDC complex. The proposed speciation scheme involved the online formation and retention of the As(III)-APDC complex at pH 3 on a SWCNTs-packed micro-column, followed by its online elution with 20% HNO_3_ and determination of As(III) by HG-AFS using a sequential flow injection manifold. Total i-As was determined by the same protocol after pre-reduction of As(V) to As(III) with thiourea; As(V) was calculated by the difference. In contrast, to effectively improve the CNT material performance for a favorable selective adsorption of As(V) in the presence of As(III), Chen et al. [26] employed multi-walled (MW) CNTs functionalized with branched cationic polyethyleneimine (BPEI) that were packed into a mini-column for online SPE of As(V) in a sequential injection system following HG-AFS detection. Adsorption of As(V) was carried out at pH 5.8, and the analyte was eluted with 0.6% NH_4_HCO_3_. By following the same procedure, total i-As was determined after the oxidation of As(III) to As(V) with H_2_O_2_.

Other nanosized adsorbents for the separation and pre-concentration of As(III,V) species from natural water samples (tap, sea, ground, underground) were also found to be useful. For example, Erdogan et al. [16] synthesized nano-zirconium dioxide-boron oxide (ZrO_2_/B_2_O_3_), called a “hybrid sorbent”, and employed it for the selective sorption of As(V) by the SPE column technique prior to its determination by HG-AAS. In this SPE procedure, As(V) ions, retained at pH 3.0, were eluted with 3 mol L^−1^ HCl and then pre-reduced to As(III) with a KI-ascorbic acid mixture and determined using HG-AAS. For total i-As, As(III) was oxidized firstly to As(V) by KMnO_4_ prior to SPE and then determined by HG-AAS; As(III) was calculated by the difference. To enhance the adsorption efficiency, Montoro Leal et al. [11] proposed magnetic nanoparticles (MNPs), i.e., ferrite (Fe_3_O_4_), which were further functionalized with [1,5-bis (2-pyridyl) 3-sulfonophenylmethylene] thiocarbonohydrazine (PSTH-MNPs) and applied for the speciation of i-As by HG-ICP-MS. This procedure was based on the retention of As(III) and As(V) at pH 4 in two knotted reactors filled with PSTH-MNPs, followed by the sequential elution of As(III) and total i-As in 7% HNO_3_-0.1% thiourea-2.8% L-cysteine medium before HG using different NaBH_4_ concentration, i.e., 0.1 for As(III) and 0.5% for total i-As and measurement by ICP-MS. The concentration of As(V) was obtained by subtracting As(III) from total i-As.

Finally, strategies involving the use of biomaterials for SPE, including baker’s yeasts (*Saccharomyces cerevisiae*) [8,37] or egg-shell membranes [21], were also proposed to speciate As species. Smichowski et al. [8] proposed a simple and sensitive method for the biosorption and pre-concentration of As(III) in the presence of As(V) in aqueous solutions using a batch system. A sample solution was combined with yeasts and 0.1 mol L^−1^ oxalic acid (acting as a reaction medium), adjusted to pH 7 and then placed in a water bath (60 °C, 30 min) to extract the As(III) form. After centrifugation, the solid phase was re-suspended in 4.0 mol L^−1^ HCl to form a slurry, the liquid phase (supernatant) was acidified to 3.5 mol L^−1^ HCl, and As(III) and As(V) were determined correspondingly in both phases by HG-ICP-AES. To overcome possible matrix effects, the method of the standard addition was used for the determination of As(III) in suspension. Under selected conditions, As(III) was almost completely (~97%) retained by the biomass, likely bounded through –SH groups of yeast proteins, while As(V) remained in the supernatant. This made it possible to determine both As species in separate phases. Several different ground water samples were analyzed following the proposed method. In contrast, Koh et al. [37] showed that As(V) was retained better than As(III) in a yeast-immobilized column. In the cited work [37], *S. cerevisiae* was covalently bound onto controlled pore glass (CPG), packed into the column and used to selectively pre-concentrate As(V) over As(III). As a result, a simple flow injection system using the yeast-immobilized column coupled online with HG and ICP-AES for sensitive determinations of As(III) and As(V) was proposed. The manifold consisted of the SPE column and a manual injector. While the CPG-yeast column (pH 7) was loaded with the sample solution, As(III) was passed through the column, and hence, could be determined by HG-ICP-OES. Moving the injector to an alternative position, elution with 3 mol L^−1^ HNO_3_ solution took place, releasing the As(V) retained on the column. The proposed method was applied for the determination of As species in herbicide, pesticide and cigarette samples. More recently, Zhang et al. [21] used a natural egg-shell membrane (ESM) as a sorptive material for SPE combined with HG-AFS to separate and determine As(V) in environmental water samples. The retention of As(V) on the ESM surface was via anion-exchange due to the presence of positively charged functional groups such as –NH_3_^+^ and –CO-NH_2_^+^. The ESM was obtained from fresh eggs and packed into a cartridge (1 g, 6 mL) by replacing its original C18 packing material. Sample solutions were adjusted to pH 11, loaded on the ESM column, and then the cartridge was washed out with water and dried. The retained As(V) was eluted with 2 mol L^−1^ HNO_3_ and measured by HG-AFS.

Regarding i-As, the application of SPE not only results in enhanced sensitivity due to the pre-concentration of the analytes; the separation of i-As also plays a crucial role in minimizing interference coming from sample matrix compounds, further improving the selectivity of i-As measurements by HG. In this way, negative effects from hydride-active methylated As species (DMA and MMA) on activity of As(III,V) under reducing conditions are overcome. Successful applications of SPE include the selective determination of total i-As using anionic exchange or nonpolar resins [41,42,43,55,79] as sorbents. In three cited works [41,42,79], silica-based strong anion exchange (SAX) cartridges were used for the determination of i-As in rice [42], various rice products [41] and seafood samples [79]. They were used for offline SPE separation of i-As from DMA and MMA, followed by HG and spectrometric detection of the sum of As(III) and As(V). Species such as As(III), As(V), DMA and MMA were extracted with dilute HNO_3_-H_2_O_2_ solutions (0.06–0.1 mol L^−1^, 1–3%) to solubilize them and oxidize all i-As to As(V). Next, sample extracts were buffered (pH 5–7.5) and loaded onto SPE cartridges. Organic As species were washed out using an acetic acid solution (0.1–0.5 mol L^−1^), and the retained As(V) ions were back-extracted (eluted) with HCl (0.5 mol L^−1^) [42,79] or HNO_3_ (0.4 mol L^−1^) solutions [41]. Finally, As(V) was pre-reduced with a KI-ascorbic acid mixture and total i-As was measured in SPE eluates as As(III) by HG-AFS [42] or HG-AAS [41,79]. It is worth mentioning that the successful separation of i-As (as As(V)), via this SAX sorbent, was achieved through pH adjustment based on dissociation constants. Unfortunately, these methods were useless when the target analyte and matrix species had similar dissociation constants. To solve these problems, a novel method for separating i-As from other species by SPE was developed, including the chemical conversion of polar i-As to nonpolar compounds, such as AsCl_3_, which can be retained by a nonpolar resin. Accordingly, Huang et al. [43] employed a polystyrene (PS) resin to retain i-As from the matrix of rice as AsCl_3_. Arsenic species were extracted with HNO_3_ (0.02 mol L^−1^), and then sample extracts were acidified with HCl to 10 mol L^−1^, treated with thiourea to reduce As(V) into to As(III) and loaded on SPE cartridges. Then, cartridges were rinsed with HCl (10 mol L^−1^), and the retained AsCl_3_ was eluted by hydrolysis with water. Finally, HG-AFS was applied to quantify the concentration of total i-As as As(III) in SPE eluates. Similarly, Zhang et al. [55] reported online SPE using PS resin cartridges coupled with HG-AFS for the determination of i-As in a complicated and arsenosugar-rich algae matrix. However, to fully overcome any matrix interferences and improve the retention efficiency of i-As, Br^-^ ions were found to be more advantageous than Cl^-^ ones for As(III) halogenation. In the proposed procedure, As species were initially extracted with HClO_4_ (1%) by a heat-vortex technique (80 °C, 20 min). Then, thiourea, KBr and HCl were added. In the presence of these reagents, As(V) was first reduced to As(III), and then i-As (as As(III)) was converted into AsBr_3_, while the role of HCl was to maintain an acidic environment for the production of AsBr_3_ retained on the cartridge. The retained AsBr_3_ was eluted from the sorbent with water and total i-As was measured as As(III) by HG-AFS. 

Otherwise, the separation o-As from i-As by SPE can be also applied only as a sample pre-treatment before analysis [23]. In this scenario, the examined water samples were passed through a glass column filled with small, activated Al_2_O_3_ to remove o-As. Adsorbed i-As species were desorbed by 0.2 mol L^−1^ HCl, made up with water to a proper volume before subsequent analysis.

#### 5.1.3. Selective Co-Precipitation 

Arsenic species can also be selectively co-precipitated for separation. Van Elteren et al. [5] proposed a method for the determination of As(III) and As(V) in bottled mineral waters at ultratrace levels by HG-AFS coupled with a flow-injection system based on selective co-precipitation of As(III) with dibenzyldithiocarbamate (DBDTC) at low pH (pH 2). The As-DBDTC precipitate was dissolved in 0.01 mol L^−1^ NaOH and 30% H_2_O_2_, and then As(III) was determined. The pre-reduction of As(V) to As(III) with a KI-K_2_S_2_O_7_ mixture was carried out before co-precipitation; this allowed the authors to determine total i-As and As(V), calculated by the difference. 

### 5.2. Pre-Concentration and Separation Methods after HG Process

Another category of pre-concentration/separation methods is the trapping of As species hydrides in a cryogenic trap (CT) before their detection. This system (HG-CT) is a convenient approach to speciation of all hydride-active As species, i.e., inorganic and methylsubstituted, due to its pre-concentration and separation ability. Generally, non-selectively formed hydrides for various As species are first cryofocussed/trapped at liquid-N_2_ temperature in a U-shaped tube (acting as a separator of different hydrides), then sequentially released from the trap by its heating according to given boiling points (BP), i.e., AsH_3_ (−55 °C, derived from As(III,V)), CH_3_AsH_2_ (2 °C, derived from MMA) and (CH_3_)_2_AsH (35.6 °C, derived from DMA), and finally transported to atomic spectrometers. To separate arsines in the trap, various types of packings were used; however, chromatographic materials are typically applied [24,61].

The main advantage of the HG-CT system is that analyses can be performed directly or with only a minimum sample pre-treatment, minimizing the risk of species inter-conversion. In view of this, an interesting approach to the determination of i-As, DMA and MMA in baby food (porridge powders and baby meals) by HG-CT-AAS, based on a slurry sampling using 3 mol L^−1^ HCl, was proposed [48]. Moreover, complex biological matrices such as human urine, cells, tissue or blood were simply lysed and/or diluted with water before analysis [31,60,61,62,63]. These minimally pretreated samples were then directly introduced into the HG device in the form of suspensions.

A remarkable quality of the HG-CT technique for As speciation is its ability to determine methylated three- and penta- valent As species in addition to inorganic ones. Here, the differentiation between inorganic and methylated As(III)- and As(V)-species in the reaction with NaBH_4_ is based on pH-dependent selective HG of arsines of As species of respective valences. Alternatively, it can be HG in the presence or absence of pre-reduction agents (usually L-cysteine), making it possible to overcome the different sensitivities of individual As species, as observed with the pH-specific HG approach [60]. To overcome the problems associated with the low and narrow range of HCl concentrations required for HG reactions in the presence of L-cysteine, the use of buffered media, i.e., Tris-HCl, was proposed. Furthermore, this approach also ensured a selective HG for the trivalent As species without its pre-reduction. This selective HG-CT approach was successfully combined with AAS and applied for analyses of different biological materials. Matousek et al. [60] and Hernandez-Zavala et al. [61] proposed oxidation state-specific speciation of inorganic and methylated arsenicals in complex biological matrices (e.g., cell cultures or tissue homogenates) by an automated HG-CT-AAS system equipped with a multiatomizer. Arsines for As(III,V), DMA(III,V) and MMA(III,V) were pre-concentrated and separated in a cryogenic chromatographic trap. To differentiate between As(III)- and As(V)-containing arsenicals, arsines for As(III)-species were generated in the presence of a Tris-HCl buffer (pH 6). Under the same conditions, a sample pre-treatment with L-cysteine allowed the authors to generate arsines for both As(III)- and As(V)-species. Finally, the content of As(V)-species was calculated as the difference. LODs of arsenicals ranged between 0.016–0.040 μg L^−^^1^ [61]. The proposed method was further improved by the online pre-reduction step integrated with the HG-CT system using TGA for pre-reduction [62]. The applicability of this method was also demonstrated in the case of As speciation in human urine samples. Replacing AAS with more sensitive AFS or ICP-MS detectors, and employing the identical HG-CT system, LODs of As species were significantly lowered to low ng L^−^^1^ and sub ng L^−^^1^ levels. This permitted analyses to be performed of limited-size samples (e.g., tissue) or samples with an extremely low As content. Accordingly, Musil et al. [63] carried out speciation of inorganic and methyl-substituted arsenicals. i.e., As(III), i-As, DMA and MMA, in exfoliated bladder epithelial cells isolated from human urine by selective HG-CT-AFS with extremely low LODs of these As species, i.e., 0.00044, 0.00074, 0.00015 and 0.00017 μg L^−^^1^, respectively. In another work [31], the HG-CT-ICP-MS-based method was shown to be suitable for direct As speciation in whole blood and blood plasma at low exposure levels with LODs of 0 μg L^−^^1^ (i-As), 0.002 μg L^−^^1^ (MMA) and 0.001 μ L^−^^1^ (DMA). For comparison, these LODs were about an order of magnitude lower than those achievable with the HG-CT-AAS system that was used for the analysis of the same samples, i.e., 0.15 μg L^−^^1^ (i-As), 0.09 μg L^−^^1^ (MMA) and 0.07 μg L^−^^1^ (DMA). 

Although a typical HG-CT system is equipped with a U-shaped tube, improvements to this trap have also been reported. For example, Hsiung and Wang [24] proposed a novel packed cold finger trap (PCFT) packed with a chromatographic material for the determination of As(III,V), DMA and MMA in fresh water and seawater by HG-AAS with a flame-heated, quartz-tube atomizer (QTA) under species-selective HG conditions. The advantage of this PCFT module over a typical U-shaped cryogenic trap lay in its better separation of collected arsines prior to their detection. In the proposed speciation scheme, As(III) was determined using a citrate buffer (pH 6.4), and As(V) was assessed by subtracting the As(III) content from the total i-As content determined after pre-reduction of As(V) to As(III) with KI, while for DMA and MMA determination, compromised levels of chemical and instrumental parameters were selected. These parameters refer to the HCl-NaBH_4_ concentrations for effective HG reactions, the flow rate of the carrier gas (He) and the heating voltage of the PCFT device for reasonable separation between both methylated species. The achieved LODs were 0.047, 0.042, 0.0045 and 0.0063 μg L^−^^1^ for As(III), As(V), MMA and DMA, respectively. More recently, Maratta et al. [19] developed a novel methodology for As speciation in ground and cistern water samples based on selective HG and CT on a CNTs-packed column, followed by elution of adsorbed arsines with 5% HNO_3_ and their quantification by ETAAS. This speciation strategy involved the selective determination of As(III) and As(V), in addition to either inorganic (hydride-active) and organic (non-hydride-active) As fractions. As(III) was determined selectively using a citric buffer (pH 4), and As(III)+As(V) was determined after the pre-reduction of As(V) with L-cysteine and thiourea. In the case of the As organic fraction, it was decomposed into hydride-active species by UV photo-oxidation catalyzed by TiO_2_. This method yielded an enrichment factor of 60 and the LOD of As of 0.00078 μg L^−^^1^.

Interestingly, by combining two complementary techniques, i.e., HG-AAS and HG-CT-AAS, a complete experimental speciation protocol for the determination of As(III), As(V), MMA, DMA and non-hydride reactive As species at ng L^−^^1^ levels in seawater was proposed [68]. In the first step, As was pre-concentrated by collecting the hydrides of As species into a graphite furnace before AAS detection. By selecting different HG reaction conditions, it was possible to determine As(III) alone (Tris-HCl, pH7), total hydride reactive As species (after alkaline persulfate digestion), total As (after alkaline persulfate digestion submitted to an oxidative UV irradiation treatment of non-hydride reactive As species), and by the difference, non-hydride reactive As species. In the second step, hydrides of reactive As species were cryogenically trapped on a chromatographic column, followed by their sequential release and AAS determination in a heated quartz furnace. As a consequence, this system made it possible to separate and determine i-As, DMA and MMA. 

Finally, hydrides of As species can be per-evaporated (P), i.e., separated from a sample matrix by simultaneous evaporation and gas diffusion through a membrane in a single step before performing measurements. In this manifold, the reactant mixture is transported to the lower chamber of a pervaporation unit from which the generated arsines evaporate first to the air gap between the liquid and the membrane. Then, they diffuse through the membrane into an acceptor stream (located in the acceptor chamber of this pervaporation module) for subsequent detection. Pervaporation appeared to be an excellent technique for the determination of inorganic As species in complex aqueous samples, known as “dirty”, i.e., matrices containing an elevated content of organic matter or solid particles in suspension. Moreover, it allows direct analyses to be made of this kind of sample, as no prior pre-treatment such as filtration is required. Caballo-Lopez and de Castro [82] developed the HG-P-AFS method with the LOD of As of 0.42 μg L^−^^1^ to determine As(III) and As(V) in various “dirty” samples with suspended particulate matter. Selective HG was carried out by varying the pH at which arsines were generated, i.e., pH 1.3 for As(III) and 0.2 for total i-As. Arsenic hydrides were generated in the reaction with 0.5% NaBH_4_ and 6 mol L^−1^ HCl in the absence of the pre-reduction step for total i-As. The concentration of As(V) was calculated as the difference. Importantly, under optimal conditions, the interference caused by DMA and MMA was negligible. In another work [86], the HG-P method coupled with CCD spectrophotometric detection was proposed for the selective determination of As(III) and As(V) in turbid river water samples with a high content of organic carbon. As(III) was selectively determined, generating AsH_3_ in the presence of a citrate buffer (pH 4.5). Both As(III) and As(V) were determined when HG was performed under highly acidic conditions (pH < 1). The concentrations of the As species were calculated by simultaneously solving two proportional equations.

## 6. Alternative HG Technique-Electrolytic Hydride Generation

Undoubtedly, traditional HG using acid-NaBH_4_ for the species selective generation of As hydrides is the predominant method. Nevertheless, in recent years, interest in alternative techniques like electrolytic hydride generation (EcHG) has grown. The great advantage of the EcHG technique is that formation of hydride eliminates the use of the reducing NaBH_4_ reagent. In this technique, the reduction of the analyte to its hydride takes place on the cathode surface, and only acidic electrolyte solutions are required to ensure the electric current for the reduction process. Therefore, the efficiency of EcHG depends mainly on the cathode material and electrolytic current. Accordingly, Arbab-Zavar et al. [85] proposed a EcHG spectrophotometric method for the determination of As(III) and As(V) in tap water without pre-reduction of As(V). In the method, a graphite cathode was used to perform the reduction of As(III) to AsH_3_, while a Sn/Pb alloy wire cathode was used to reduce As(V) to AsH_3_. Next, species were determined at 510 nm as the As(III)-SDDC complex obtained from the reaction between AsH_3_ and silver diethyldithiocarbamate (SDDC). The proposed method resulted in LODs of 20 (As(III)) and 60 μg L^−^^1^ (As(V)). Similarly, Li et al. [78] selectively determined i-As species by EcHG-AAS using a glassy carbon cathode in a 0.06 mol L^−1^ H_2_SO_4_ catholyte medium. Differentiation between As(III) and As(V) was based on the different HG efficiencies attained by controlling electrolytic currents at 0.6 and 1.0 A, respectively. Concentrations of both species were calculated from slopes of calibration curves with LODs of 0.2 μg L^−^^1^ for As(III) and 0.5 μg L^−^^1^ for As(V). The method was successfully applied to speciate soluble i-As in Chinese medicines. Yang et al. [22] reported that the EcHG behavior of As(III), As(V), DMA and MMA could be changed at the modified graphite electrode (GE) by –SH modifiers including L-cysteine (Cys) and glutathione (GSH). The authors proposed a four-step analysis approach for the determination of all four As species in rice and natural water (river, lake, rain) samples by EcHG-AFS. Accordingly, As(III) was reduced on the GSH/GE at an applied current of 0.4 A. As(III), and As(V) generated AsH_3_ on the Cys/GE at an applied current of 0.6 A. Measurements of As(III) and DMA were done on Cys/GE at two applied currents. Finally, the total As was determined after the reduction on Cys/GE at an applied current of 2.0 A. Under optimal conditions, LODs ranged between 0.10–0.25 μg L^−^^1^. Lu et al. [58] demonstrated the application of a chemically modified carbon paste electrode (CMCPE) in EcHG to detect (ultra)trace amounts of As species. Given this, the authors developed a procedure for the selective determination of As(III) and total As in Chinese herbal medicines by EcHG-AFS. In the procedure, As(III), As(V), MMA and DMA were first selectively reduced to AsH_3_ at an applied current of 1.0 A on Cys/CMCPE. Then, under the same conditions, total As was determined after the pre-reduction of As(V), DMA, and MMA to As(III) with L-cysteine. Compared to traditional GE, the CMCPE provided better sensitivity; with CMCPE-AFS, LODs were successfully reduced to 0.095 (As(III)) and 0.087 (total As) μg L^−^^1^.

## 7. Conclusions 

In terms of toxicological investigations, knowledge of As species is crucial to understanding the potentially harmful effects associated with exposure to this element. HPLC-ICP-MS is undoubtedly the most popular coupled method for speciation analyses of As, because it provides a complete and sophisticated picture of species eluted from one injection of a sample, quantified at a (ultra)trace level. Nevertheless, as shown in this review, current research’ interest is more focused on developing robust and reliable alternative methods obviating chromatographic separation. Non-chromatographic approaches to As speciation, based on the use of simple instrumentation which is available in most of laboratories (like atomic absorption or fluorescence detectors), are simpler, faster and economically friendly. They additionally provide degrees of sensitivity which are of the same order of magnitude or even better (related to possible species separation improving their pre-concentration) than those with hyphenated traditional chromatographic techniques using ICP-MS detection. In this sense, speciation achieved by selective HG prior to spectrometric detection is one of the most effective tools for distinguishing among four major toxic As species, i.e., As(III), As(V), DMA and MMA, or differentiating these toxic (hydride-active) species from non-toxic (non-hydride-active) arsenicals. In this way, this non-chromatographic approach gives sufficient information about As speciation for appropriate risk evaluations. Different physical-chemical properties of hydride-active As species (e.g., their volatility, redox potential) and the possibility of controlling experimental parameters such as pH, reaction medium, reactant concentration, the presence of additives, temperature, etc., make it possible to develop species-selective non-chromatographic strategies for the speciation of trace amounts of different As species in a wide range of environmental, food and biological matrices. Furthermore, HG combined with various pre-concentration/separation approaches to accomplish As speciation serves as an excellent tool for the determination of As species at ultratrace levels. There is a belief that accurate (precise and true) methods for As speciation, which could be used for routine analyses of different samples, both with simple and complex matrices, will continue to develop. 

The possibility of effectively speciating As using HG and atomic spectrometry gives rise to a willingness to further improve this technique, focused on reagent consumption, reduction, miniaturization and automation (employing various online flow systems). Simultaneously, as alternatives to chemical HG techniques, electrochemical (Ec)HG, for example, will be extensively studied and developed in the future, in an attempt to augment conventional HG for the determination and speciation of hydride forming elements. 

## Figures and Tables

**Table 1:** The concentrations of As species in different samples obtained by non-chromatographic methods based on the hydride generation technique ^a^.

Matrix	As(III), μg L^−1^	As(V), μg L^−1^	i-As, μg L^−1^	DMA, μg L^−1^	MMA, μg L^−1^	o-As ^b^, μg L^−1^
**Liquid Matrices**						
***Waters***						
drinking	0.17–4.7 [2,3,4]	0.05–63.9 [2,3,4,5]				
ground	0.080–395 [4,6,7,8,9,10,11]	0.01–312 [4,6,7,8,9,10,11]	0.31–308 [12,13,14]			
underground	1.25–1016 [3,15,16]	0.97–554.3 [3,15,16]		1.4–2.8 [3]	0.8–1.2 [3]	
tap	0.015–12.7 [4,9,16,17,18,19]	0.050–35 [4,9,15,16,17,18,19,20,21]				0.30–0.80 [19]
river	0.235–1.4 [4,10,20,22]	0.186–2.22 [4,10,20,21,22]	1.79–2.05 [14]			
lake	0.11–0.95 [10,20,23,24]	0.03–1.30 [10,20,21,22,23,24]	3.80 [14]	0.007 [24]		
sea	0.03–2.2 [3,10,11,17,24,25]	0.17–19.8 [3,10,11,17,24,25]		0.15 [24]	0.15–0.19 [3]	
waste	0.345 [4]	0.052–0.957 [4,21]				
rain		1.52 [26]	5.16 [26]			
snow		2.04 [26]	3.60 [26]			
***Beverages***						
wine	1.3–21.3 [27,28]					
tea	0.3–14.4 [29]	56–59.6 [29]				11.4–23.3 [29]
fruit juices	0.3–3.9 [30]	0.12–6.6 [30]				
***Biological Samples (human fluids/tissue)***						
serum	0.604–0.838 [4]	1.087–3.010 [4]				
urine	0.548–3.142 [4]	0.410–1.334 [4]				
blood/blood plasma			15.8–19.2 [31]	13.4–34.8 [31]	13.5–30.6 [31]	
**Solid Matrices**						
Matrix	As(III), ng g^−1^	As(V), ng g^−1^	i-As, ng g^−1^	DMA, ng g^−1^	MMA, ng g^−1^	o-As ^b^, ng g^−1^
***Environmental***						
soil	5.2–8.1 ^c^ [32]	16.0–20.4 ^c^ [32]				
phosphate rocks	2.1–3.9 µg g^−1^ [33]		5.2–20.0 µg g^−1^ [33]			
airborne particulate matter	2.7–10.5 ng m^−3^ [34]		3.8–20 ng m^−3^ [34]			
***Industrial***						
glass	13.6–395 µg g^−1^ [35]	10.6–1205 µg g^−1^ [35]				
***Agricultural Agents***						
phosphate fertilizers	2.6–7.5 µg g^−1^ [33,36]		11.79–69.02 µg g^−1^ [33,36]			
pesticide	0.90 µg g^−1^ [37]	0.81 µg g^−1^ [37]				
herbicyde		1.47 µg g^−1^ [37]				
***Food***						
rice	22–248 [22,38,39,40]	5–76 [22,38,39,40]	30–600 [41,42,43,44,45,46]	4.2–67.3 [22,39,40]	2.2–38.1 [22,39]	12.2–112.2 [45]
rice products	12.3–52.7 [47]	1.4–29.6 [47]	20–570 [41,42,43,44,45,46,47,48]	15–89 [48]	2.9–5.3 [48]	
wheat semolina	55 [39]	25 [39]		1.9 [39]	1.5 [39]	
milk	1.5–5.9 [49]	2.1–8.1 [49]				
mushrooms	81–624 [50]	59–380 [50]				
chard	89.2–90.6 [51]	14.2–15.3 [51]		4.1–4.3 [51]	3.5–3.7 [51]	
aubergine	20.6–20.9 [51]	61.0–61.9 [51]		1.1–1.2 [51]	1.2 [51]	
***Marine Organism***						
fish	80.3–230 [52]	108–310 [52]		510–1310 [52]	490–780 [52]	9.4–16 ^a,c^ µg g^−1^ [52]
seaweed			0.05–57.5, µg/g [44,53,54,55]			
***Pharmaceuticals***						
TCM (herbs)	22.8 ^c^ [56]	145.1 ^c^ [56]				116.4 ^b,d^ [56]
herbaceous plant	0.030–8.32 µg g^−1^ [57,58]	0.050–4.59 µg g^−1^ [57]	1.08–6.91 µg g^−1^ [58]			0.040, µg g^−1^ [57]
dietary supplements	25–93 [59]	58–201 [59]				

i-As: the inorganic tri- and pentavalent As species (As(III) and As(V)). TCM: traditional Chinese medicines. ^a^ Concentration ranges based on reported results in works cited in this review. ^b^ Total organic As species concentrations (DMA+MMA) calculated as the difference between total As (determined after digestion) and i-As concentrations. ^c^ Water soluble fraction. ^d^ As non-toxic, i.e., unreactive As forms toward HG (mainly AsB) calculated as the difference between total As (determined after digestion) and the hydride-active toxic As species, i.e., the sum of As(III)+As(V)+DMA+MMA.

**Table 2:** Non-chromatographic procedures for individual speciation, i.e., selective determination of As(III) and As(V).

Matrix	Species	Sample Preparation ^a^	Speciation Approach	Detection	LOD ^b^, μg L^−^^1^	Ref.
**Redox Property of NaBH_4_ (kinetic-dependent reduction reaction between NaBH_4_ and As(IIII,V) forms)**						
natural waters	As(III) As(V)	direct analysis	*I. Selective As(III):* S: not acidified, A: 10 M HCl, R: 0.05% NaBH_4_ *II. i-As (as As(III)):* S: L-cysteine, A: 10 M HCl, 0.6% NaBH_4_	HG-ICP-OES	1.0	[83]
water CRMs (TMDA-54.3, CASS-4)	As(III) As(V)	direct analysis	*I. Selective As(III):* S: 4 M HCl, A: 6 M HCl, 0.2% NaBH_4_ *II. i-As (as As(III)):* S: KI-ascorbic acid–4 M HCl, A: 6 M HCl, 0.2% NaBH_4_	HG-AFS	0.05	[80]
natural waters	As(III) As(V)	direct analysis	*I. Selective As(III):* S: 0.1 M HCl, 4.0% NaBH_4_ *II. i-As (as As(III)):* S: KI-ascorbic acid–1 M HCl, 4.0% NaBH_4_	HG-AAS	1.2	[15]
natural waters	As(III) As(V)	direct analysis	*I. Selective As(III):* A: 1.5 M HCl, R: 0.5% NaBH_4_ *II. i-As (as As(III)):* A: 9.0 M HCl 3.0% NaBH_4_	HG-AAS	As(III): 0.1 As(V): 0.06	[20]
ground water	As(III) As(V)	direct analysis	*I. Selective As(III):* S: 0.1 M HCl, A: 2 M HCl, 0.035% NaBH_4_, *II. i-As (as As(III)):* S: KI-ascorbic acid–2.6 M HCl, A: 2 M HCl, R: 0.2% NaBH_4_,	HG-AAS	As(III): 1.4 As(V): 0.6	[12]
rice	As(III) As(V)	MAE (0.14 M HNO_3_)	*I. Selective As(III):* S: 0.14 M HNO_3_, A: 10 M HCl, 0.1% NaBH_4_ *II. i-As (as As(III)):* S: 0.14 M HNO_3_–KI-ascorbic acid–1.2 M HCl, 10 M HCl, R: 0.1% NaBH_4_	HG-AAS	As(III): 0.07 As(V): 0.14	[38]
rice products	As(III) i-As	HP (0.28 M HNO_3_)	*I. Selective As(III):* S: 0.28 M HNO_3_, A: 10 M HCl, 0.1% NaBH_4_ *II. i-As (as As(III)):* S: 0.28 M HNO_3_–KI-ascorbic acid–1.9 M HCl, 10 M HCl, R: 0.1% NaBH_4_	HG-AAS	As(III): 0.08 i-As: 0.14	[47]
**Specific HG from As(III) and As(V) under controlled pH conditions or different reaction media**						
drinking water	As(III) As(V)	direct analysis	*I. Selective As(III):* S: Tris-HCl buffer (pH 7.2), R: 3.0% NaBH_4_ *II. i-As (as As(III)):* S: Tris-HCl (pH 7.2)–TGA, R: 3.0% NaBH_4_	HG-AFS	As(III): 0.027 As(V): 0.036	[2]
ground water	As(III) As(V)	direct analysis	*I. Selective As(III):* S: not acidified, A: 0.1 M citric acid, R: 0.6% NaBH_4_ *II. i-As (as As(III)):* S: KI–6.5 M HCl, A: 10 M HCl, R: 0.6% NaBH_4_	HG-AAS	0.4	[6]
ash and soil CRMs (NIST 1633b GBW07302 GBW07311)	As(III) As(V)	two-step sequential LE (H_2_O, 1 mM phosphate buffer)	*I. Selective As(III):* S: citric buffer (pH 4.5), A: 10% HCl, R: 0.3% NaBH_4_; optionally: S: 2% HCl, A: 2% HCl, R: 0.3% NaBH_4_ *II. i-As (as As(III)):* S: KI-ascorbic acid–HCl, A: 10% HCl, R: 0.3% NaBH_4_; optionally: S: 0.46% TGA in sample, A: 10% HCl, R: 0.3% NaBH_4_	HG-AAS	As(III): 0.07 As(V): 0.06	[72]
soil	As(III) As(V)	four-step sequential LE (H_2_O, 0.6 M KH_2_PO_4_, 1% HCl, 1% NaOH)	*I. Selective As(III):* S: 0.1 M citric acid, R: 1.0% NaBH_4_ *II. i-As (as As(III))* S: 0.1 M citric acid–L-cysteine, 1.0% NaBH_4_	HG-AFS	As(III): 0.11 As(V): 0.07	[32]
glass	As(III) As(V)	dissolution (24% HF)	*I. Selective As(III):* S: citric buffer (pH 4.5), R: 1.0% NaBH_4_ *II. i-As (as As(III)):* S: 6.0 M HCl, R: 1.0% NaBH_4_	HG-AAS		[35]
airborne particulate matter	As(III) i-As	SS (4 M HCl)	*I. Selective As(III):* S: citric buffer (pH 7.1), A: 2 M HCl, R: 2.15% NaBH_4_ *II. i-As (as As(III)):* S: KI-ascorbic acid–1.8 M HCl, A: 2 M HCl, R: 2.15% NaBH_4_	HG-AAS	0.1	[34]
phosphate fertilizer and rock	As(III) i-As	SS (6 M HCl)	*I. Selective As(III):* S: citric buffer (pH 7.1), A: 2 M HCl, R: 2.15% NaBH_4_ *II. i-As (as As(III)):* S: KI-ascorbic acid–2 M HCl, A: 2 M HCl, R: 2.15% NaBH_4_	HG-AAS	0.1	[33]
phosphate fertilizer	As(III) i-As	US (0.35% Triton X-114 + 6.5 M HNO_3_)	*I. Selective As(III):* S: citric buffer (pH 4.5), A: 10% HCl, R: 0.4% NaBH_4_ *II. i-As (as As(III)):* S: thiourea, A: 10% HCl, R: 0.4% NaBH_4_	HG-AAS	As(III): 0.029 i-As: 0.022	[36]
wine	As(III) i-As	EtOH evaporation	*I. Selective As(III):* S: 8 M HCl, A: 9 M HCl, R: 0.2% NaBH_4_, *II. i-As (as As(III)):* S: KI– 8 M HCl, A: 9 M HCl, R: 0.2% NaBH_4_	HG-AAS	0.1	[27]
plankton	As(III) As(V)	MAE (H_2_O)	*I. Selective As(III):* S: citric buffer (pH 4.5), A: 2% HCl, R: 0.1% NaBH_4_ *II. i-As (as As(III)):* S: citric buffer (pH 4.5)–L-cysteine, A: 2% HCl, R: 0.1% NaBH_4_	HG-MF-AAS	2.0	[70]
**Species-selective respond toward two different reducing conditions (As speciation based on systems of linear independent equations)**						
milk	As(III) As(V)	SS (*aqua regia*)	1.2% NaBH_4_–3.5 M HCl without and after pre-reduction with KI-ascorbic acid-hydroxylamine hydrochloride in 10.8 M HCl	HG-AAS	As(III): 0.0081 As(V): 0.0103	[49]
mushrooms	As(III) As(V)	US (1 M H_3_PO_4_-0.1% Triton X-100 + 0.1% EDTA)	0.7% NaBH_4_–3.5 M HCl without and after pre-reduction with KI-ascorbic acid in 9 M HCl	HG-AFS	6.5 ng g^−^^1 c^	[50]
TCM (herbs)	As(III) As(V)	LE (H_2_O)	1.0% KBH_4_–1 M HCl without and after pre-reduction with KI-thiourea in 2 M HCl	HG-AFS	0.0797	[56]
tea	As(III) As(V)	brewing (H_2_O)	*I. Selective As(III):* S: 0.1 M citric acid–5% HCl, A: 5% HCl, R: 5% KBH_4_ *II. i-As (as As(III)):* S: thiourea-ascorbic acid–5% HCl, A: 5% HCl, R: 5% KBH_4_	HG-AFS	As(III): 0.0070 As(V): 0.0095	[29]
natural waters	As(III) As(V)	direct analysis	*I.* S: 0.7 M HCl, R: 0.7% NaBH_4_ *II.* S: L-cysteine–0.1 M HCl, R: 0.7% NaBH_4_	HG-AFS	As(III) 0.013 As(V): 0.015	[17]
**Specific HG from As(III) and As(V) in the presence of masking reagents**						
herbaceous plant	As(III) As(V)	LE (1% HCl)	*I. Selective As(III):* S: 8-hydroxyquinoline–10% HCl, R: 2.5% NaBH_4_ *II. i-As (as As(III)):* 8-hydroxyquinoline–KI-ascorbic acid–10% HCl, R: 2.5% NaBH_4_	HG-AFS		[57]
dietary suplements	As(III) As(V)	SS (50% HCl)	*I. Selective As(III):* S: 8-hydroxyquinoline–1% HCl, R: 1.0% KBH_4_ *II. i-As (as As(III)):* S: 8-hydroxyquinoline–KI–5% HCl, R: 1.0% KBH_4_	HG-AAS	As(III): 0.080 As(V): 0.089	[59]

EtOH: ethanol. HG-AAS: hydride generation atomic absorption spectrometry. HG-AFS: hydride generation atomic fluorescence spectrometry. HG-MF-AAS: metallic furnace hydride generation atomic absorption spectrometry. HG-ICP-OES: hydride generation inductively-coupled plasma optical emission spectrometry. HP: heating in water bath on hot plate. i-As: the inorganic tri- and pentavalent As species (As(III) and As(V)). LE: solvent extraction. M: mol L^−1^. MAE: microwave-assisted extraction. S: sample solution. A: additional acid (carrier) solution. R: reductant solution. SS: slurry sampling. TCM: traditional Chinese medicines. TGA: thioglycolic acid. US: ultrasonication in an ultrasound water bath. WB: water bath. ^a^ Detailed information about sample preparation procedures described in point 4. ^b^ For As(V) or i-As as As(III) without or after previous pre-reduction step. ^c^ In the original sample, taking into account the amount of sample and the final dilution employed in the proposed procedure.

**Table 3:** Non-chromatographic procedures for operational speciation, i.e., i-As determination (total As(III) and As(V)).

Matrix	Species	Sample Preparation ^a^	Speciation Procedures	Detection	LOD ^b^, μg L^−1^	Ref.
rice, seaweed	i-As (as As(V))	MAE (1–2% HNO_3_–3% H_2_O_2_)	S: 3% H_2_O_2_, A: 5 M HCl, R: 2% NaBH_4_	HG-ICP-MS	0.006	[44]
rice	i-As (as As(III))	US (*aqua regia*)	S: 1.25 M *aqua regia*–KI-ascorbic acid–3 M HCl, A: 10 M HCl, R: 1% NaBH_4_	HG-ICP-OES	0.28 ng g^−1^	[45]
rice	i-As (as As(III))	MAE (0.28 M HNO_3_)	S: 0.28 M HNO_3_–KI-ascorbic acid–3 M HCl, A: 1.2 M HCl, R: 0.1% NaBH_4_	HG-AAS	16 ng g^−1 c^	[46]
seafood, seaweed	i-As (as As(V))	MAE (2% HNO_3_–3% H_2_O_2_)	S: 3% H_2_O_2_, A: 8 M HCl, R: 2% NaBH_4_	HG-ICP-MS	0.01	[53]
seaweed	i-As (as As(V))	MAE (2% HNO_3_–3% H_2_O_2_)	S: 3% H_2_O_2_, A: 5 M HCl, R: 2% NaBH_4_	HG-ICP-MS	0.06	[54]

HG-AAS: hydride generation atomic absorption spectrometry. HG-ICP-OES: hydride generation inductively-coupled plasma optical emission spectrometry. HG-ICP-MS: hydride generation inductively-coupled plasma mass spectrometry. i-As: the inorganic tri- and pentavalent As species (As(III) and As(V)). M: mol L^−1^. MAE: microwave-assisted extraction. S: sample solution. A: additional acid (carrier) solution. R: reductant solution. US: ultrasonication in an ultrasound water bath. ^a^ Detailed information about sample preparation procedures described in point 4. ^b^ For As(V) as As(III) without or after previous pre-reduction step. ^c^ In the original sample taking into account the amount of sample and the final dilution employed in the proposed procedure.

**Table 4:** Non-chromatographic speciation and fractionation protocols for the determination of various As species by HG.

Matrix	Species	Sample Preparation ^a^	Speciation Procedures	Detection	LOD ^b^, μg L^−1^	Ref.
cereals, fish, vegetables	As(III) As(V) DMA MMA t-As_toxic_ t-As_non-toxic_	US (3 M HNO_3_ or 1 M H_3_PO_4_–0.1% Triton X-114 + 0.1% EDTA)	*-Individual speciation:* *I:* 2 M HCl, 1.4% NaBH_4_ (maximum for DMA, MMA) *II:* 4 M HCl, 1.4% NaBH_4_ (intermediate for all species) *III:* 3.5 M HCl, 1.2% NaBH_4_ (maximum for As(III,V) *IV:* KI-ascorbic acid–3.5 M HCl, 1.2% NaBH_4_ (with pre-reduction As(V) and MMA to As(III)) *-Operational speciation:* t-As_toxic_ and t-As_non-toxic_ fractions as difference between total As content (T-As) determined after complete sample digestion and sum of As(III,V), DMA and MMA species (t-As_toxic_)	HG-AFS	As(III): 0.62-3.1 ng g^−1 c^ As(V): 0.9-3.0 ng g^−1 c^ DMA: 1.5-1.8 ng g^−1 c^ MMA: 0.6-5.4 ng g^−1 c^	[39,51,52] [52]
rice	As(III) As(V) DMA MMA	US (1% HNO_3_)	*I:* 0.06 M citric acid (maximum for As(III)+DMA) *II:* K_2_S_2_O_8_–0.06 M citric acid (maximum for DMA) *III:* L-cysteine-ascorbic acid–0.06 M citric acid (maximum for As(III)+As(V)+DMA) *IV:* thiourea-ascorbic acid–5% HCl (pre-reduction of As(V), DMA and MMA to As(III)) A: 1.6 M HCl–citrate buffer (pH 4.8), R: 2% KBH_4_	HG-AFS	As(III): 0.21 μg kg^−1^ As(V): 0.52 μg kg^−1^ DMA: 0.65 μg kg^−1^ MMA: 0.9 μg kg^−1^	[40]
ground water	As(III) As(V) DMA	direct analysis	*I. As(III):* S: oxalate buffer (pH 4-4.5), R: 0.6% NaBH_4_ *II. As(III)+As(V):* S: KI, A: 6 M HCl, R: 0.6% NaBH_4_ *III. DMA:* S: L-cysteine, A: 1.5 M HCl, R: 0.6% NaBH_4_	HG-AAS	As(III): 0.1 As(III,V): 0.1 DMA: 0.19	[7]
natural waters	As(III) As(V) DMA MMA	direct analysis	*I. As(III)+As(V)+MMA:* S: KI–0.1 M HCl, A: 1 M HCl, R: 0.6% NaBH_4_ *II. As(III)+DMA:* S: 0.1 M HCl, A: 6 M CH_3_COOH, R: 0.6% NaBH_4_ *III. As(III)+As(V)+DMA:* S: KI–0.1 M HCl, A: 6 M CH_3_COOH, R: 0.6% NaBH_4_ *IV. As(III)+As(V)+DMA+MMA:* S: KI–0.1 M HCl, A: 1 M tartaric acid, R: 0.6% NaBH_4_	HG-AAS	0.1	[3]
wine	As(III) As(V) DMA MMA	5-10 dilution	*I. As(III):* S: citrate buffer (pH 5.1), R: 0.6% NaBH_4_ *II. As(III)+DMA:* S: 0.2 M CH_3_COOH, R: 0.6% NaBH_4_ *III. As(III)+As(V):* S: KI–8 mol/L HCl, R: 0.2% NaBH_4_ *IV. As(III)+As(V)+DMA+MMA:* S: L-cysteine–0.01 M HCl, R: 0.6% NaBH_4_	HG-AAS	As(III): 0.4 As(V): 0.3 DMA: 0.3 MMA: 0.3	[28]
fish	As(III) As(V) DMA MMA	MAE (H_2_O:MeOH, 1:4 v:v))	*I. As(III):* S: citrate buffer (pH 5.2), R: 0.45% NaBH_4_ *II. As(III)+DMA:* S: 0.2 M CH_3_COOH, R: 0.45% NaBH_4_ *III. As(III)+As(V):* S: KI–7 M HCl, R: 0.2% NaBH_4_ *IV. As(III)+As(V)+DMA+MMA:* S: L-cysteine–0.05 M HCl, R: 0.45% NaBH_4_	HG-AAS	As(III): 3.5μg kg^−1c^ As(V): 5.1 μg kg^−1c^ DMA: 4.8 μg kg^−1c^	[81]
plant, sediment	As(III) As(V) DMA	US (6 M HCl)	*I. As(III):* S: 2% HCl, R: 1.2% NaBH_4_ S: 15% HNO_3_, R: 1.2% NaBH_4_ *II. As(III)+As(V):* S: 10% HNO_3_, R: 1.2% NaBH_4_ *III. As(III)+DMA:* S: 2% HNO_3_, R: 1.2% NaBH_4_	HG-AFS	As(III): 3.1 As(V): 5.7 DMA: 3.8	[75]
drinking water	As(III) As(V) DMA MMA i-As o-As	direct analysis	*I. As(III):* S: citrate buffer (pH 5.2), R: 1.0% NaBH_4_ *II. As(III)+DMA:* S: acetate buffer (pH 4.5), R: 1.0% NaBH_4_ *III. As(III)+As(V)+MMA:* S: KI-ascorbic acid–3 M HCl, A: 10 M HCl, R: 1.0% NaBH_4_ *IV. DMA+MMA:* S: L-cysteine, A: 2 M HCl, R: 1.0% NaBH_4_ *V. As(III)+As(V)+DMA+MMA:* S: L-cysteine, A: 10 M HCl, R: 1.0% NaBH_4_	HG-ICP-OES		[71]
soil and sediment CRMs	As(III) As(V) DMA MMA	WB (0.05 M EDTA)	*I. As(III)+DMA+MMA:* S: 0.02 M EDTA (pH 5-6), *II. As(III)+As(V)+DMA+MMA:* S: 0.02 M EDTA (pH 5-6) + KI-ascorbic acid/HCl followed by neutralization to pH 5-7 A: 1.2 M HCl, R: 0.6% NaBH_4_	HG-AAS	0.2 mg kg^−1 c^	[77]
ground water	i-As DMA	direct analysis	*I. i-As (As(III)+As(V)):* S: KI-ascorbic acid–2 M HCl, A: 3 M HCl, R: 2.2% NaBH_4_ *II. As(III)+As(V)+DMA (UV photo-oxidation):* S: K_2_S_2_O_8_, A: 3 M HCl, R: 2.2% NaBH_4_	HG-AFS	As(III,V): 0.09 DMA: 0.47	[13]
water, soil, sediments	As(III) As(V) DMA	direct analysis/US	*I. As(III):* S: not acidified, A: 2% HCl, R: 0.5% NaBH_4_ *II. As(III)+DMA (UV photo-reduction):* S: ZnO–8% formic acid, 2% HCl, R: 0.5% NaBH_4_ *III. As(III)+As(V)+DMA (UV photo-reduction):* S: ZnO–8% formic acid, 10% HCl, R: 0.5% NaBH_4_	HG-AFS	As(III): 3.20 As(V): 3.86 DMA: 6.68	[76]

HG-AAS: hydride generation atomic absorption spectrometry. HG-AFS: hydride generation atomic fluorescence spectrometry. HG-ICP-OES: hydride generation inductively-coupled plasma optical emission spectrometry. HG-ICP-MS: hydride generation inductively-coupled plasma mass spectrometry. i-As: the inorganic tri- and pentavalent As species (As(III) and As(V)). M: mol L^−1^. MAE: microwave-assisted extraction. o-As: the organic, i.e., methylated pentavalent As species (DMA and MMA). MeOH: methanol. S: sample solution. A: additional acid (carrier) solution. R: reductant solution. US: ultrasonication in an ultrasound water bath. WB: water batch. ^a^ Detailed information about sample preparation procedures described in point 4. ^b^ Pentavalent As species as As(III) without or after previous pre-reduction step. ^c^ In the original sample taking into account the amount of sample and the final dilution employed in the proposed procedure.

## Supplementary Materials

The following are available online, Table S1: Pre-concentration/separation methods to accomplish the speciation of inorganic As species by HG-based non-chromatographic techniques.
