# Supplementary material for: Non-Chromatographic Speciation of As by HG Technique—Analysis of Samples with Different Matrices
Source: Molecules. 2020 Oct 26;25(21):4944. doi: 10.3390/molecules25214944 (PMC7663061; doi:10.3390/molecules25214944)
Supplement: Supplementary file 1 [file molecules-25-04944-s001.pdf]

Table 1S.

Pre-concentration/separation methods to accomplish the speciation of inorganic As species by HG-based non-chromatographic techniques.

Matrix	Species	Procedure	Detection	LOD[a], μg L^{-1}	EF[b]	Ref.
CPE						
natural waters	As(III) As(V)	Selective reaction between As(III) and Pyronine B in presence of SDS at pH 10 followed by extraction of As(III)-Pyronine B complex into Triton X-114. Total i-As was extracted after pre-reduction of As(V) to As(III) with $Na_2S_2O_3$; As(V) calculated by difference.	HG-AAS	0.008	25.7	[18]
Lake water	As(III) As(V)	Selective formation of As(III)-APDC (at pH 4.6) and As(V)-molybdate (at pH 2.4) complexes followed by extraction into Triton X-114. As species determined separately.	HG-AFS	As(III): 0.009 As(V): 0.012	As(III): 9.3 As(V): 7.9	[23]
DLLME						
Fruit juices	As(III) As(V)	Selective reaction between As(III) and APDC at pH 3 followed by As(III)-APDC complex extraction into CCl_4 in dispersive medium (methanol). Total i-As was extracted after pre-reduction of As(V) to As(III) with $Na_2S_2O_3$ (pH 1.7-1.8); As(V) calculated by difference.	HG-AFS	1.2		[30]
ATPS						
tap water	As(III)	Selective extraction of As(III)-APDC at pH 6 using ATPS (electrolyte and polymer based) composed of L64 (copolymers) + Na_2SO_4 + H_2O.	HG-ICP-OES	0.20 (μg kg^{-1})		[84]
co-precipitation						
drinking water	As(III) As(V)	Selective co-precipitation of As(III) with DBDTC at pH 2. After separation, the co-precipitated As(III)-DBDTC was dissolved with NaOH-H_2O_2. Total i-As was co-precipitated after pre-reduction of	HG-AFS	0.05		[5]

		As(V) to As(III) with KI-$K_2S_2O_7$; As(V) calculated by difference.				
SPE						
natural waters	As(V)<br>As(III)	Selective adsorption of As(V) at pH 6 on aluminum hydroxide gel followed by dissolution in concentrated HCl and pre-reduction with thiourea-ascorbic acid. Total i-As was adsorbed after pre-oxidation of As(III) to As(V) with $KMnO_4$; As(III) calculated by difference.	HG-AFS	0.003	167[c]	[14]
natural waters	As(III)<br>As(V)	Selective reaction between As(III) and APDC with next retention of the As(III)-APDC complex on the column packed with cigarette filter. Total i-As was extracted after pre-reduction of As(V) to As(III) with thiourea-ascorbic acid; As(V) calculated by difference. Eluent: 1.68 M HCl.	HG-AFS	0.0074	25.6	[9]
sea water	As(III)<br>As(V)	Selective formation of As(III)-APDC complex followed by its sorption on the mini-column contains PTFE particles as sorbent. Total i-As was extracted after pre-reduction of As(V) to As(III) with thiourea; As(V) calculated by difference. Eluent: 2 M HCl.	HG-AAS	As(III): 0.02<br>As(V): 0.03	20.4	[25]
natural waters	As(V)<br>As(III)	Selective sorption of As(V) at pH 3.5 on the G-COOH (column technique). Retained As(V) was eluted with 0.5 M NaOH and then pre-reduced to As(III) with KI-ascorbic acid. Total i-As was extracted after pre-oxidation of As(III) to As(V) with $KMnO_4$; As(III) calculated by difference.	HG-AAS	0.0021	50.3	[4]
natural waters	As(III)<br>As(V)	Selective formation and retention of the As(III)-APDC complex at pH 3 on the micro-column packed with SWCNTs followed by elution with 20% HNO_3. Total i-As was extracted after pre-reduction of As(V) to As(III) with thiourea; As(V) calculated by difference.	HG-AFS	As(III): 0.0038<br>As(V): 0.0043	25.4	[10]

natural waters	As(V)<br>i-As	As(V) was selectively separated from As(III) at pH 5.8 with the MWCNTs-BPEI packed mini-column. Retained As(V) was desorbed using 0.6% NH_4HCO_3. Total i-As was extracted after pre-oxidation of As(III) to As(V) with H_2O_2.	HG-AFS	As(V): 0.014	16.3	[26]
natural waters	As(V)<br>As(III)	As(V) sorbs selectively at pH 3 on the nano ZrO_2/B_2O_3 hybrid sorbent (column technique) and then desorbs by 3 M HCl followed by pre-reduction with KI-ascorbic acid. Total i-As was extracted after pre-oxidation of As(III) to As(V) with $KMnO_4$. As(III) calculated by difference.	HG-AAS	0.00925	20	[16]
natural waters	As(III)<br>As(V)	Selective sorption of As(III) and As(V) at pH 4 in two knotted reactors filled with PSTH-MNPs followed by elution using 7% HNO_3–0.1% thiourea–2.8% L-cysteine and selective HG for As(III) and i-As. As(V) calculated by difference.	HG-ICP-MS	As(III): 0.0027<br>As(V): 0.0032	As(III): 1.9<br>As(V): 2.1	[11]
natural waters	As(III)<br>As(V)	Selective biosorption of As(III) into the baker's yeast (*Saccharomyces cerevisiae*). At pH 7 As(III) was accumulated by the yeast cells, while As(V) remained in solution. After separation, the solid phase was prepared to be slurry for determination. As species determined in separate phases.	HG-ICP-AES	As(III): 0.1<br>As(V): 0.5	7[c]	[8]
herbicide, pesticide, cigarette	As(V)<br>As(III)	Selective biosorption of As(V) using column packed with yeast (*Saccharomyces cerevisiae*) immobilized on the CPG. At pH 7 As(V) sorbs selectively, whereas As(III) was not retained and passed directly through the column. Both As(III) and As(V) determined separately. Eluent: 3 M HNO_3.	HG-ICP-AES			[37]

ground water	As(V)	Selective retention of As(V) on the ESM at pH 11. Eluent: 2 M HNO_3.	HG-AFS	0.001	33.3	[21]
rice products,<br>marine organism,<br>rice	i-As (as As(V))	Strong anion exchange (SAX) cartridges for selective sorption of i-As coexisting with o-As at pH 6. Eluent: 0.4 M HNO_3 [41] or 0.5 M HCl [42,79].	HG-AAS<br>HG-AFS		0.02 mg kg^{-1}<br>0.08 mg kg^{-1}<br>1.3 ng g^{-1}	[41]<br>[79]<br>[42]
marine algae	i-As (as As(III))	Polystyrene (PS) resin cartridge for selective sorption of $AsBr_3$ [55] or $AsCl_3$ [43] coexisting with As-sugars and/or o-As under strongly acidic condition. Eluent: H_2O	HG-AFS		3.0 ng g^{-1}<br>1.1ng g^{-1}	[55]<br>[43]

APDC: ammonium pyrrolidinedithiocarbamate. ATPS: liquid-liquid extraction of aqueous two-phase systems. DBDTC: dibenzyldithiocarbamate. DLLME: dispersive liquid-liquid microextraction. SDS: sodium dodecyl sulfate. CPE: cloud point extraction. CPG: controlled pore glass. ESM: eggshell membrane. G-COOH: carboxylated nanoporous grapheme. HG-AAS: hydride generation atomic absorption spectrometry. HG-AFS: hydride generation atomic fluorescence spectrometry. HG-ICP-AES: hydride generation inductively-coupled plasma atomic emission spectrometry. HG-ICP-OES: hydride generation inductively-coupled plasma optical emission spectrometry. HG-ICP-MS: hydride generation inductively-coupled plasma mass spectrometry. i-As: the inorganic tri- and pentavalent As species (As(III) and As(V)). M: mol L^{-1}. MWCNTs-BPEI: multi-walled carbon nanotubes functionalized with branched cationic polyethyleneimine. o-As: the organic, i.e. methylated pentavalent As species (DMA and MMA). PSTH-MNPs: magnetic ferrite (Fe_3O_4) nanoparticles functionalized with [1,5-bis (2-pyridyl) 3-sulfonophenylmethylene] thiocarbonohydrazine. PTFE: polytetrafluororoethylene. SWCNTs: single-walled carbon nanotubes.

[a] For As(V) as As(III) without or after previous pre-reduction step.

[b] Enrichment factor: the ratio of the slopes of the calibration curves for As with and without pre-concentration.

[c] Pre-concentration factor: the ratio of initial to final volume of enriched solution after pre-concentration.
